# ESGO/ESTRO/ESP Guidelines for the management of patients with cervical cancer – Update 2023*

**DOI:** 10.1136/ijgc-2023-004429

**Published:** 2023-04-26

**Authors:** David Cibula, Maria Rosaria Raspollini, François Planchamp, Carlos Centeno, Cyrus Chargari, Ana Felix, Daniela Fischerová, Daniela Jahnn-Kuch, Florence Joly, Christhardt Kohler, Sigurd Lax, Domenica Lorusso, Umesh Mahantshetty, Patrice Mathevet, Raj Naik, Remi A Nout, Ana Oaknin, Fedro Peccatori, Jan Persson, Denis Querleu, Sandra Rubio Bernabé, Maximilian P Schmid, Artem Stepanyan, Valentyn Svintsitskyi, Karl Tamussino, Ignacio Zapardiel, Jacob Lindegaard

**Affiliations:** 1 Department of Obstetrics and Gynecology, Charles University, First Faculty of Medicine, Prague, Czech Republic; 2 General University Hospital in Prague, Prague, Czech Republic; 3 University Hospital Careggi, Firenze, Italy; 4 Clinical Research Unit, Institut Bergonie, Bordeaux, France; 5 Department of Palliative Medicine, University of Navarra, Pamplona, Spain; 6 Service d’Oncologie Radiothérapie, Hôpital Universitaire Pitié Salpêtrière, Paris, France; 7 Instituto Portugues de Oncologia de Lisboa Francisco Gentil EPE, Lisboa, Portugal; 8 Universidade Nova de Lisboa, Lisboa, Portugal; 9 Department of Internal Medicine, Medical University of Graz, Graz, Austria; 10 François Baclesse Centre de Lutte Contre le Cancer, Caen, France; 11 Asklepios Clinic Altona, Hamburg, Germany; 12 Asklepios Comprehensive Tumor Center, Hamburg, Germany; 13 Hospital Graz II, Graz, Austria; 14 Johannes Kepler Universitat Linz, Linz, Austria; 15 Fondazione Policlinico Universitario A.Gemelli IRCCS, Rome, Italy; 16 Catholic University of Sacred Heart, Rome, Italy; 17 Homi Bhabha Cancer Hospital and Research Centre, Visakhapatnam, Andhra Pradesh, India; 18 CHUV, Lausanne, Switzerland; 19 Northern Gynaecological Oncology Centre, Gateshead, UK; 20 Radiotherapy, Erasmus MC Cancer Centre, Rotterdam, The Netherlands; 21 University Medical Center, Rotterdam, The Netherlands; 22 Vall d'Hebron Institute of Oncology (VHIO), Barcelona, Spain; 23 Hospital Universitari Vall d'Hebron, Barcelona, Spain; 24 European Institute of Oncology IRCCS, Milan, Italy; 25 Department of Obstetrics and Gynecology, Lund University Hosptial, Lund, Sweden; 26 Skåne University Hospital Lund, Lund, Sweden; 27 University Hospitals Strasbourg, Strasbourg, France; 28 Medical Oncology, Clinica Universidad de Navarra, Pamplona, Spain; 29 Department of Radiation Oncology, Medical University of Vienna, Wien, Austria; 30 Gynecologic Oncology, Nairi Medical Center, Yerevan, Armenia; 31 National Cancer Institute, Kiev, Ukraine; 32 Medical University of Graz, Graz, Austria; 33 Gynecologic Oncology, La Paz University Hospital, Madrid, Spain; 34 Aarhus University Hospital, Aarhus, Denmark

**Keywords:** Cervical Cancer, Pathology, Radiation, Surgical Oncology

## Abstract

In 2018, the European Society of Gynecological Oncology (ESGO) jointly with the European Society for Radiotherapy and Oncology (ESTRO) and the European Society of Pathology (ESP) published evidence-based guidelines for the management of patients with cervical cancer. Given the large body of new evidence addressing the management of cervical cancer, the three sister societies jointly decided to update these evidence-based guidelines. The update includes new topics to provide comprehensive guidelines on all relevant issues of diagnosis and treatment in cervical cancer.

To serve on the expert panel (27 experts across Europe) ESGO/ESTRO/ESP nominated practicing clinicians who are involved in managing patients with cervical cancer and have demonstrated leadership through their expertise in clinical care and research, national and international engagement, profile, and dedication to the topics addressed. To ensure the statements were evidence based, new data identified from a systematic search was reviewed and critically appraised. In the absence of any clear scientific evidence, judgment was based on the professional experience and consensus of the international development group. Before publication, the guidelines were reviewed by 155 independent international practitioners in cancer care delivery and patient representatives.

These updated guidelines are comprehensive and cover staging, management, follow-up, long-term survivorship, quality of life and palliative care. Management includes fertility sparing treatment, early and locally advanced cervical cancer, invasive cervical cancer diagnosed on a simple hysterectomy specimen, cervical cancer in pregnancy, rare tumors, recurrent and metastatic diseases. The management algorithms and the principles of radiotherapy and pathological evaluation are also defined.

## Introduction

Cervical cancer is a major public health problem, ranking as the fourth most common cause of cancer incidence and mortality in women worldwide. There are geographical variations in cervical cancer that reflect differences particularly in the prevalence of human papillomavirus (HPV) infection and inequalities in access to adequate screening and treatment.^1^ Cervical cancer is uncommon in Europe but still remains the most frequent cause of cancer death in middle-aged women in Eastern Europe.^2^ Other epidemiologic risk factors associated with cervical cancer are notably a history of smoking, oral contraceptive use, early age of onset of coitus, number of sexual partners, history of sexually transmitted disease, certain autoimmune diseases, and chronic immunosuppression. Squamous cell carcinomas account for approximately 80% of all cervical cancers and adenocarcinoma accounts for approximately 20%. The WHO recently launched a global initiative to scale up preventive, screening, and treatment interventions relying on vaccination against HPVs, screening and treatment of detected cervical pre-invasive and invasive lesions, and offering the best possible curative care to women diagnosed with invasive cancer.^3^

As part of its mission to improve the quality of care for women with gynecological cancers across Europe, in 2018 the European Society of Gynecological Oncology (ESGO) jointly with the European Society for Radiotherapy and Oncology (ESTRO) and the European Society of Pathology (ESP) published evidence-based guidelines to improve the management of patients with cervical cancer within a multidisciplinary setting.^4–6^ Given the large body of new evidence addressing the management of cervical cancer, the three sister societies jointly decided to update these evidence-based guidelines and to include new topics in order to provide comprehensive guidelines on all relevant issues of diagnosis and treatment in cervical cancer. These guidelines are intended for use by gynecological oncologists, general gynecologists, surgeons, radiation oncologists, pathologists, medical and clinical oncologists, radiologists, general practitioners, palliative care teams, and allied health professionals.

## Responsibilities

Even though our aim is to present the highest standard of evidence in an optimal management of patients with cervical cancer, ESGO, ESTRO, and ESP acknowledge that there will be broad variability in practices between the various centers worldwide. Moreover, there will also be significant differences in infrastructure, access to medical and surgical technology, and also training, medicolegal, financial, and cultural aspects that will affect the implementation of any guidelines. These guidelines are a statement of evidence and consensus of the multidisciplinary development group regarding their views and perspective of currently accepted approaches for the management of patients with cervical cancer. Any clinician applying or consulting these guidelines is expected to use independent medical judgment in the context of individual clinical circumstances to determine any patient’s care or treatment. These guidelines make no representations or warranties of any kind whatsoever regarding their content, use, or application and disclaim any responsibility for their application or use in any way.

## Methods

**Figure 1:**
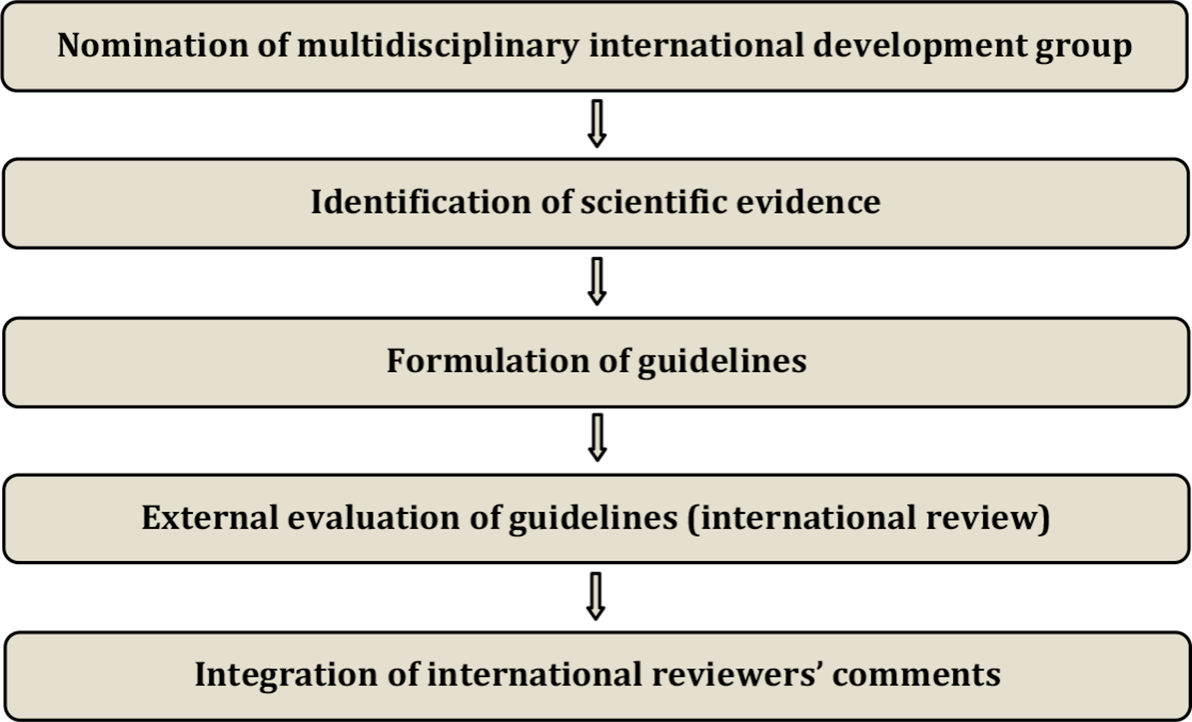
Guideline development process.

The guidelines were developed using a five-step process defined by the ESGO Guideline Committee (see Figure 1). The strengths of the process include creation of a multidisciplinary international development group, use of scientific evidence and international expert consensus to support the guidelines, and use of an international external review process (physicians and patients). This development process involved three meetings of the international development group, chaired by Professor David Cibula (First Faculty of Medicine, Charles University and General University Hospital, Prague, Czech Republic), Professor Jacob Christian Lindegaard (Aarhus University Hospital, Aarhus, Denmark), and Professor Maria Rosaria Raspollini (University of Florence, Florence, Italy).

**Figure 2:**
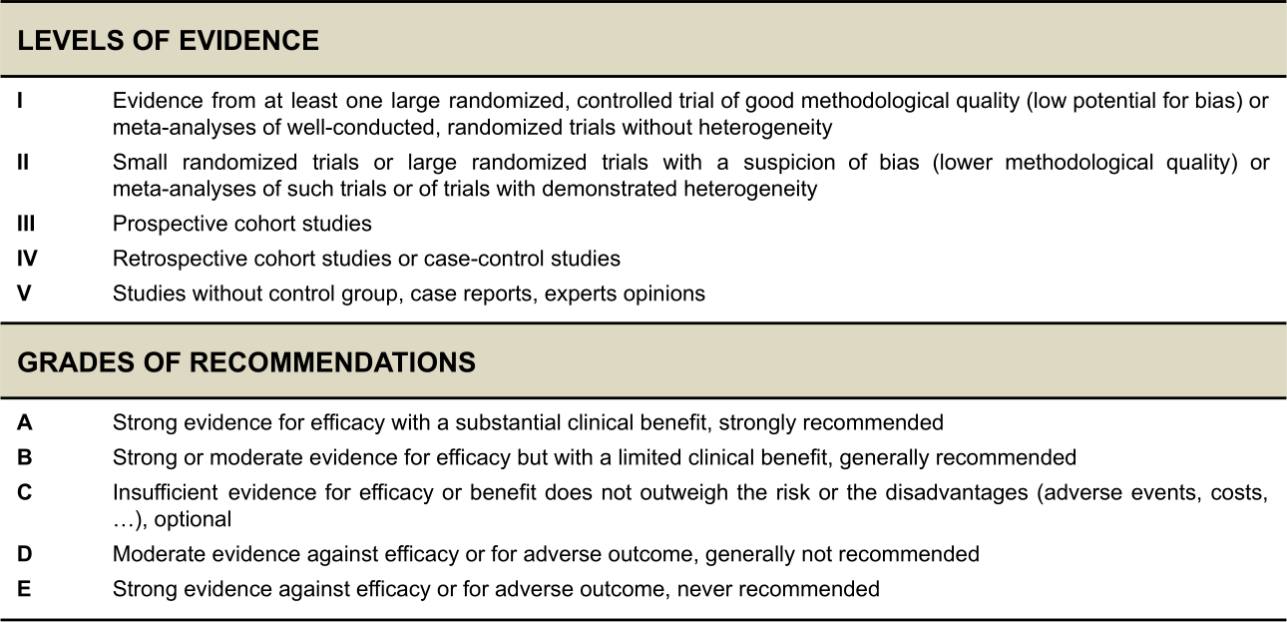
Levels of evidence and grades of recommendations.

To serve on the expert panel, ESGO/ESTRO/ESP nominated practicing clinicians who are involved in managing patients with cervical cancer and have demonstrated leadership through their expertise in clinical care and research, national and international engagement and profile as well as dedication to the topics addressed. The objective was to assemble a multidisciplinary development group and it was therefore essential to include professionals from relevant disciplines (gynecological oncology and gynecology, medical, clinical and radiation oncology, pathology) to contribute to the validity and acceptability of the guidelines. To ensure that the statements were evidence based, the current literature was reviewed and critically appraised. A systematic, unbiased literature review of relevant studies published between January 2017 and March 2022 was carried out using the MEDLINE database (see Online Supplemental File 2). The literature search was limited to publications in English. Priority was given to high-quality systematic reviews, meta-analyses, and randomized controlled trials, but studies of lower levels of evidence were also evaluated. The search strategy excluded editorials, letters, and in vitro studies. The reference list of each identified article was reviewed for other potentially relevant articles. Based on the collected evidence and clinical expertise, the international development group drafted guidelines for all the topics. The updated guidelines were retained if they were supported by a sufficiently high level of scientific evidence and/or when a large consensus among experts was obtained. An adapted version of the “Infectious Diseases Society of America–United States Public Health Service Grading System was used to define the level of evidence and grade of recommendation for each of the recommendations^7^ (see Figure 2). In the absence of any clear scientific evidence, judgment was based on the professional experience and consensus of the international development group.

10.1136/ijgc-2023-004429.supp2 Supplementary data

ESGO/ESTRO/ESP established a large multidisciplinary panel of practicing clinicians who provide care to patients with cervical cancer to act as independent reviewers for the updated guidelines. These reviewers were selected according to their expertise, had to be still involved in clinical practice/research, and were from different European and non-European countries to ensure a global perspective. Patients with cervical cancer were also included. The independent reviewers were asked to evaluate each recommendation according to its relevance and feasibility in clinical practice (only physicians), so that comprehensive quantitative and qualitative evaluations of the updated guidelines were completed. Patients were asked to evaluate qualitatively each recommendation (according to their experience, personal perceptions, etc.). Evaluations of the external reviewers (n=155) were pooled and discussed by the international development group to finalize the guidelines’ updating process. The list of the 155 external reviewers is available in Online Supplemental File 2.

## Guidelines

The guidelines detailed in this article cover staging, management, follow-up, long-term survivorship, quality of life and palliative care. Management includes fertility sparing treatment, early and locally advanced cervical cancer, invasive cervical cancer diagnosed on a simple hysterectomy (SH) specimen, cervical cancer in pregnancy, rare tumors, recurrent and metastatic diseases. A summary of evidence supporting the guidelines is included in Online Supplemental File 1, available online.

10.1136/ijgc-2023-004429.supp1 Supplementary data

### General Recommendations

- Centralization of care in specialized centers and referral network is encouraged [IV, B].
- Treatment planning should be made on a multidisciplinary basis (generally at a tumor board meeting as defined in the ESGO quality indicators) and based on the comprehensive and precise knowledge of prognostic and predictive factors for oncological outcome, side effects, and quality of life [IV, A].
- Patients should be carefully counseled on the suggested treatment plan and potential alternatives, including risks and benefits of all options [V, A].
- Treatment should be undertaken by a dedicated team of specialists in the diagnosis and management of cervical cancers [IV, A].
- Enrollment of patients with cervical cancer in clinical trials is encouraged [V, B].

### Staging

#### TNM Classification and FIGO Staging

- Patients with cervical cancer should be staged according to the TNM classification and the International Federation of Gynaecology and Obstetrics (FIGO) staging should also be documented [IV, A].
- Systematic documentation and integration of the results from clinical examination, pathology and imaging including multidisciplinary team discussions of disparate findings is recommended [IV, A].
- The method used to determine tumor status (T), lymph node (LN) status (N), and systemic status (M) should be noted (clinical, imaging, pathological) [IV, A].
- Lymph node (LN) metastases should be classified according to the TNM classification [IV, A].

#### Prognostic Factors

- Systematic documentation of the following major tumor-related prognostic factors is recommended [II, A]:
  - TNM and FIGO stage, including a maximum tumor size, detailed description of extracervical tumor extension (including uterine corpus involvement) and nodal involvement (eg, total number, location, size, and metabolic activity).
  - Pathological tumor type including HPV status (see principles of pathological evaluation).
  - Depth of cervical stromal invasion and a minimum thickness of uninvolved cervical stroma
  - Margin status (ectocervical, endocervical, radial/deep stromal and vaginal cuff)
  - Presence or absence of lymphovascular space involvement (LVSI).
  - Presence or absence of distant metastases.

#### Local Clinical and Radiological Diagnostic Work-up

- Pelvic examination and biopsy±colposcopy are mandatory to diagnose cervical cancer [II, A].
- Pelvic magnetic resonance imaging (MRI) is mandatory for initial assessment of pelvic tumor extent and to guide treatment options (optional for T1a tumor with free margins after conization). Endovaginal/transrectal ultrasonography is an option if performed by a properly trained sonographer [II, A].
- Cystoscopy or proctoscopy are not routinely recommended [IV, D].

#### Nodal/Distant Diagnostic Work-up

- In early stages managed primarily by surgery, surgical/pathological staging of pelvic lymph node (PLN) is the standard criterion to assess the prognosis and to guide treatment (except for T1a1 and T1a2 without LVSI) [III, A].
- In locally advanced cervical cancer (T1b3 and higher (except T2a1) or in early-stage disease with suspicious LN on imaging), positron emission tomography-computed tomography (PET-CT), or chest/abdomen computed tomography (CT scan) (if PET-CT is not available) is recommended for assessment of nodal and distant disease [III, B].
- PET-CT is recommended before chemoradiotherapy (CTRT) with curative intent [III, B].
- Para-aortic LN dissection (PALND), at least up to inferior mesenteric artery, may be considered in locally advanced cervical cancer with negative para-aortic LN on imaging for staging purposes [IV, C].
- Equivocal extrauterine disease should be considered for biopsy to avoid inappropriate treatment [IV, B].

### Management of T1a Disease

#### Diagnosis of T1a Disease

- Diagnosis of T1a cancer should be based on a conization (or excision) specimen examined by an expert pathologist with accurate measurement of depth of invasion, margin status, coexisting pathology, and reliable assessment of LVSI [IV, B].
- Loop or laser conization is preferable to cold-knife conization in women wanting to preserve fertility. Care should be taken to provide an intact (unfragmented) specimen with minimal thermal artifact. The cone specimen should be oriented for the pathologist [IV, B].
- Surgical margins of the cone specimen should be clear of both invasive and preinvasive disease (except for low-grade intraepithelial lesion) [IV, B].

#### Management of T1a1 Disease

- Management of patients with T1a1 disease should be tailored to the individual depending on age, desire for fertility preservation, histological type, and the presence or absence of LVSI [III, B].
- In case of positive margins (except for low-grade intraepithelial lesion in ectocervix), a repeat conization should be performed to rule out more extensive invasive disease [IV, B].
- LN staging is not indicated in T1a1 LVSI-negative patients but can be considered in T1a1 LVSI-positive patients. Sentinel lymph node (SLN) biopsy (without additional PLN dissection (PLND)) is recommended in this situation [IV, B].
- Conization can be considered a definitive treatment as hysterectomy does not improve the outcome [IV, C].
- Radical surgical approaches such as radical hysterectomy, trachelectomy or parametrectomy represent overtreatment and should not be performed for patients with T1a1 disease [IV, D].
- Patients with T1a1 adenocarcinoma who have completed childbearing should be offered SH [IV, B].

#### Management of T1a2 Disease

- Conization (with clear margins) alone or SH is an adequate treatment for patients with T1a2 disease [IV, B].
- Parametrial resection is not indicated [IV, D].
- SLN biopsy (without additional PLND) can be considered in LVSI-negative patients but should be performed in LVSI-positive patients [IV, B].
- Patients with T1a2 adenocarcinoma who have completed childbearing should be offered SH [IV, B].

### Management of T1b1, T1b2, and T2a1 Tumors

#### General Recommendations

- Treatment strategy should aim to avoid combining radical surgery and radiotherapy because of the high morbidity induced by the combined treatment [IV, A].

### Negative LN on Radiological Staging - Surgical Treatment

- Radical surgery by a gynecological oncologist is the preferred treatment modality. Laparotomy is the standard approach for all procedures which include radical parametrectomy [I, A].
- Minimally invasive approach may be considered only in low risk tumors (<2 cm and free margins after conization), in high-volume centers experienced in performing radical hysterectomy with minimally invasive surgery, which meet the ESGO quality criteria for surgery, if the patient agrees after comprehensive discussion about current evidence [IV, C].
- LN assessment should be performed as the first step of surgical management [IV, A]. Minimally invasive surgery is an acceptable approach for LN staging [IV, B].
- SLN biopsy before pelvic lymphadenectomy should be performed. Indocyanine green is the preferred technique [III, A]. A combination of blue dye with radiocolloid is an alternative technique [IV, B].
- Intra-operative assessment of LN status (evaluated by frozen section) is recommended. Sentinel nodes from both sides of the pelvis and/or any suspicious LN should be sent for intra-operative assessment [III, A].
- If any LN involvement is detected intraoperatively, further PLND and radical hysterectomy should be avoided. Patients should be referred for definitive CTRT [III, A]. PALND at least up to inferior mesenteric artery may be considered for staging purposes [IV, C].
- After SLN biopsy, if SLN are negative on frozen section, a systematic pelvic lymphadenectomy should be performed as the standard LN staging [III, A].
- If SLN is negative bilaterally in the pelvic level I area (below iliac bifurcation) LN dissection can be limited to level I [IV, B].
- If SLN is not detected on either side, LN dissection should include on that particular pelvic side the removal of lymphatic tissue from all traditional regions including obturator fossa, external iliac regions, common iliac regions, and presacral region [III, A].
- After frozen section, all SLN should be processed according to pathological protocol for ultrastaging (see the principles of pathological evaluation) [III, A].
- The type of radical hysterectomy (extent of parametrial resection, type A-C2) should be based on the presence of prognostic risk factors identified preoperatively such as tumor size, maximum stromal invasion, and LVSI, which are used to categorize patients at high, intermediate, and low risk of treatment failure. A complete description of the template used for radical hysterectomy should be present in the surgical report. The 2017 modification of the Querleu-Morrow classification is recommended as a tool [IV, A].
- Ovarian preservation should be discussed with women in reproductive age with squamous cell carcinoma, can be considered in HPV-associated adenocarcinoma and is not recommended for HPV-independent adenocarcinomas. Opportunistic bilateral salpingectomy should be performed if ovaries are preserved. Ovarian transposition should be discussed upfront with the patient and individualized according to risk balance [IV, A].
- If a combination of risk factors is known at diagnosis, which would require an adjuvant treatment, definitive CTRT and brachytherapy (BT) should be considered without previous radical pelvic surgery [IV, A].

#### Negative LN on Radiological Staging – Alternative Treatment Options

- Definitive CTRT and image-guided brachytherapy (IGBT) represent an alternative treatment option [IV, B].
- Neoadjuvant chemotherapy (NACT) or CTRT followed by surgery are not recommended [IV, D].

#### Adjuvant Treatment After Radical Surgery

- Adjuvant radiotherapy should be considered in the intermediate risk group (combination of risk factors at final pathology such as tumor size, LVSI, and depth of stromal invasion) [IV, A].
- When an adequate type of radical hysterectomy has been performed in intermediate risk group patients, observation is an alternative option, especially in teams experienced in this approach [IV, B].
- Adjuvant CTRT is indicated in the high-risk group (see principles of radiotherapy) [IV, A]:
  - metastatic involvement of PLN (macrometastases pN1 or micrometastases pN1(mi)) on final pathologic assessment.
  - positive surgical margins (vagina/parametria/paracervix).
  - parametrial involvement.
- Additional BT boost as part of adjuvant CTRT can be considered in cases with vaginal and/or parametrial positive disease (see principles of radiotherapy) [IV, B].
- Adjuvant treatment may be considered also if only isolated tumor cells are detected in SLN, although its prognostic impact remains uncertain [IV, C].

### Fertility Sparing Treatment

**Figure 3:**
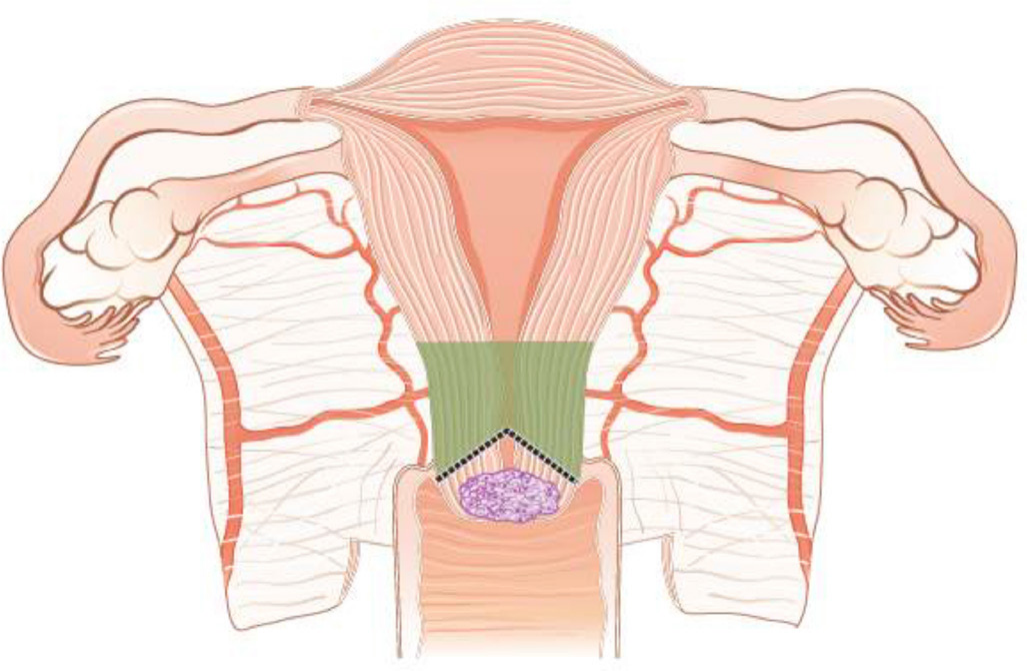
Conization.

**Figure 4:**
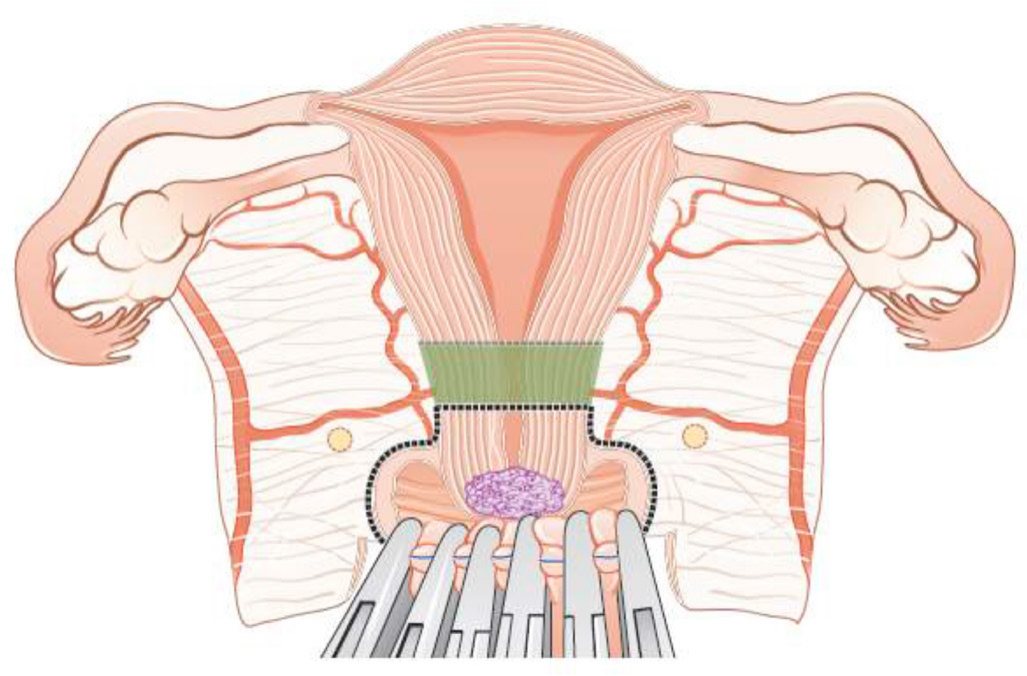
Simple trachelectomy.

**Figure 5:**
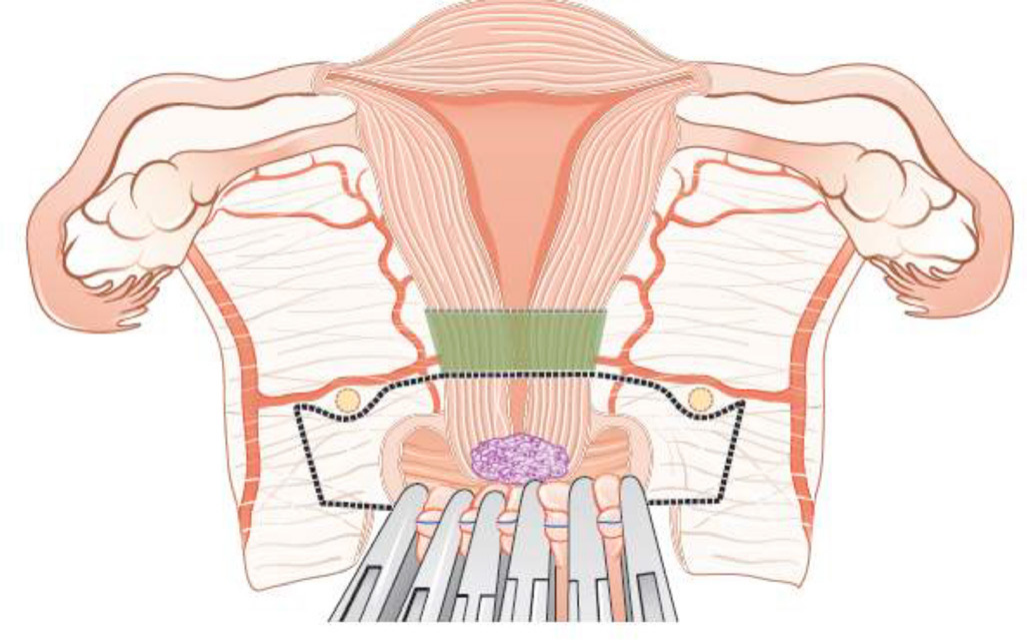
Radical (vaginal) trachelectomy.

**Figure 6:**
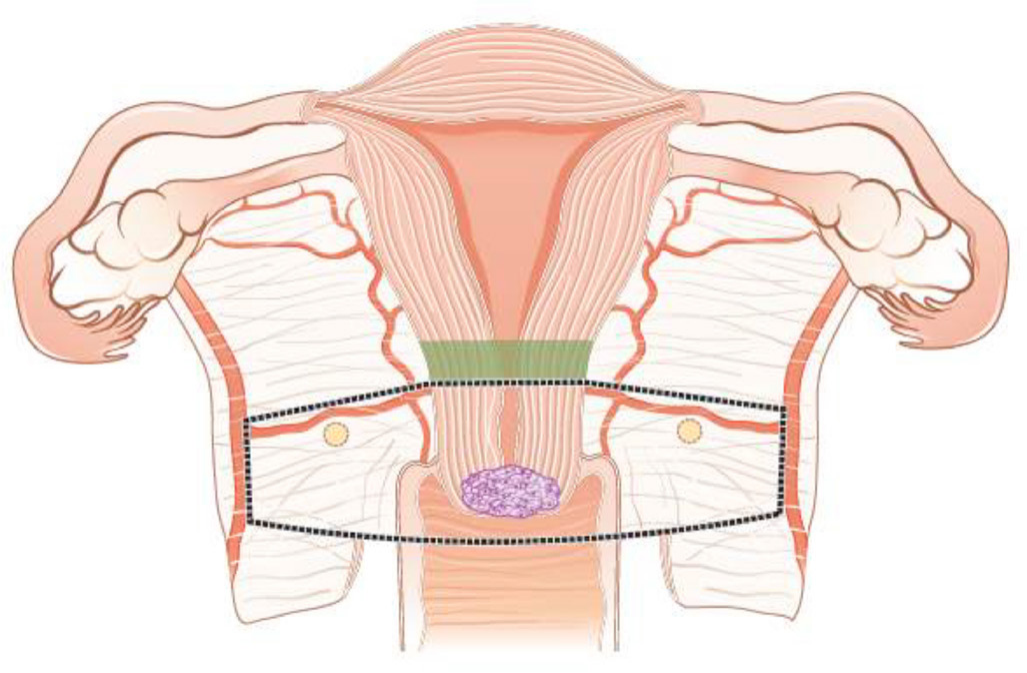
Abdominal radical trachelectomy.

**Figure 7:**
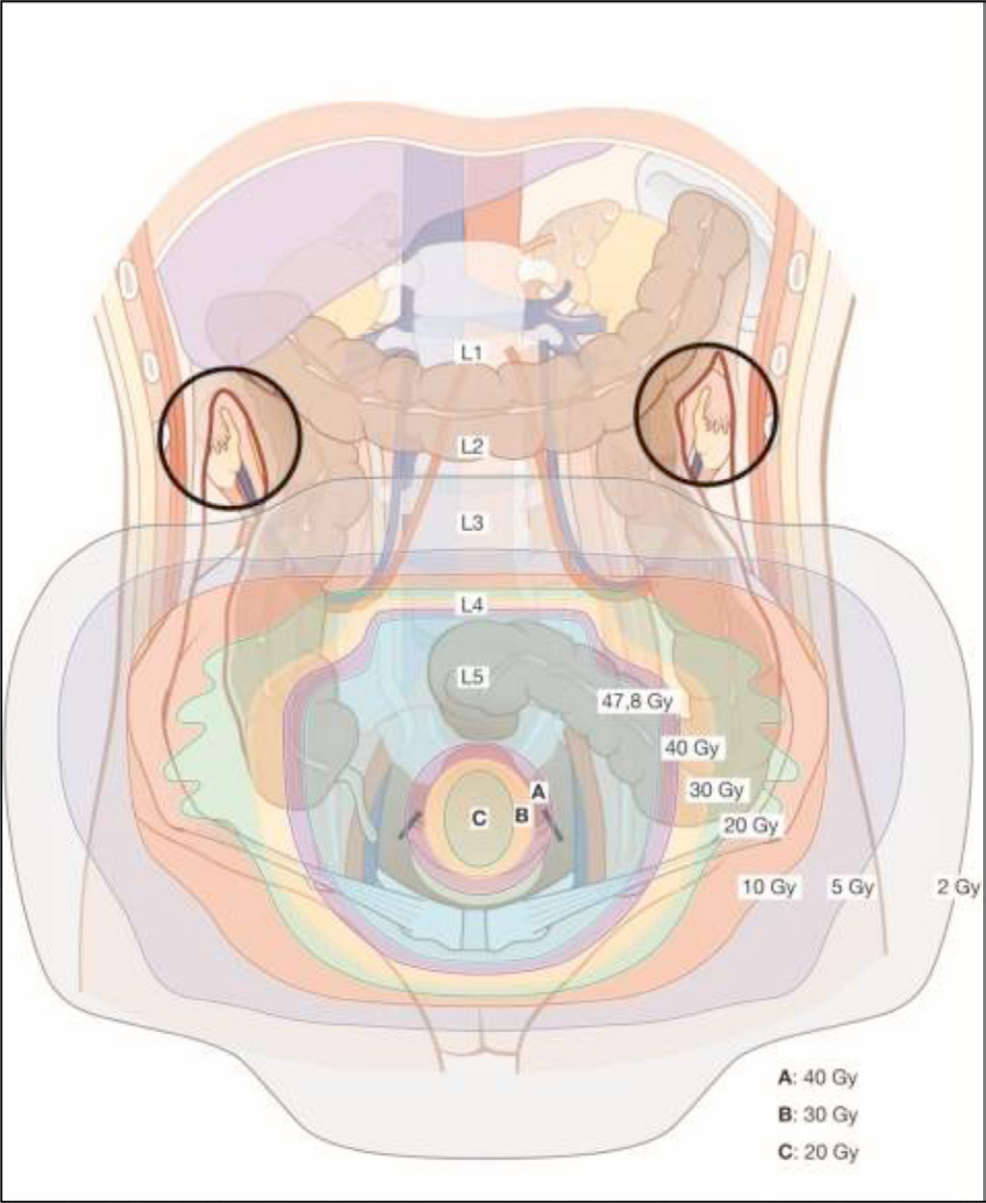
Ovarian transposition.

- Fertility sparing therapy is an oncologically valid alternative to radical hysterectomy for young patients with cervical cancer <2 cm (squamous cell carcinoma and HPV-related adenocarcinoma) who want to preserve the option to have children. Before initiating fertility sparing therapy, consultation at an onco-fertility center and discussion in a multidisciplinary tumor board is recommended [III, B].
- Counseling of eligible patients should encompass the oncologic and obstetric risks related to this type of management as well as the risk of fertility sparing therapy abandonment if there are positive resection margins or LN involvement [III, A].
- Fertility-sparing treatment should be performed exclusively in gynaecological-oncological centers with comprehensive expertise in all types of these surgical procedures [IV, A].
- Fertility-sparing treatment should not be recommended for uncommon and rare histological types/subtypes of cervical cancer with aggressive behavior including neuroendocrine carcinomas, HPV-independent adenocarcinomas and carcinosarcomas [V, D].
- For patients who consider fertility sparing therapy, prognostic factors, clinical staging, and preoperative work-up do not differ from those not considering fertility sparing therapy (see above). Pelvic MRI and/or expert sonography are mandatory imaging tests to measure the non-involved cervical length (upper tumor free margin) and the remaining (after cone biopsy) cervical length [III, A].
- Negative PLN status is the precondition for any fertility sparing therapy. Therefore, PLN staging (SLN) should always be the first step in each fertility-sparing therapy procedure. Identification of SLN and its ultrastaging is highly recommended. Any intraoperative suspicious LN (apart from SLN) should also be removed. If SLN cannot be detected on either pelvic side, a systematic pelvic lymphadenectomy should be performed on that side. Intraoperative assessment of LN status is highly recommended. All SLN from both sides of the pelvis and any suspicious LN should be sent for frozen section. LN staging is not indicated in T1a1 LVSI negative [III, A].
- In case of intraoperatively proven PLN involvement, fertility-sparing surgery should be abandoned and patients should be referred for CTRT and BT [IV, B]. PALND, at least up to inferior mesenteric artery, may be considered for staging purposes [IV, C]. Ovarian transposition cannot be recommended in N1 status [IV, D].
- The specific goal of fertility-sparing surgery must be resection of invasive tumor with adequate free margins and preservation of the upper part of the cervix [IV, A]. Intraoperative frozen section is a feasible way of assessing the upper resection margin [IV, C].
- LN staging follows the principles of management of early stages [III, B].
- Fertility sparing procedures comprise of conization (see Figure 3), simple trachelectomy (see Figure 4), radical (vaginal) trachelectomy (see Figure 5), abdominal radical trachelectomy (see Figure 6) [III, B].
- Conization and simple trachelectomy are adequate fertility sparing procedures in patients with T1a1 and T1a2 tumors, regardless of LVSI status [IV, B].
- Conization or simple trachelectomy are adequate fertility sparing procedures for T1b1, LVSI negative tumors. Radical trachelectomy is still an option [IV, B].
- Radical trachelectomy (type B) should be performed in patients with cervical cancer T1b1, LVSI-positive. In patients without deep stromal involvement and with a high probability of adequate endocervical tumor free margins, simple trachelectomy can be considered [III, B].
- Intraoperative placement of permanent cerclage should be performed during simple or radical trachelectomy [IV, B].
- Fertility sparing therapy for patients with tumors greater than 2 cm is significantly associated with a higher risk of recurrence and should not be considered as a standard treatment. The risk of recurrence must be comprehensively discussed with the patient. NACT followed by radical vaginal trachelectomy and abdominal radical trachelectomy or cone has been described for fertility sparing treatment in patients with tumors >2 cm. PLN staging should be performed before starting NACT to confirm tumor-free LN. The optimal number of chemotherapy cycles, chemotherapy regimen as well as extent of cervical resection following NACT, are still a matter of debate [IV, B].
- In more advanced cases, various fertility preservation proposals such as ovarian transposition (see Figure 7), oocyte-, embryo- or ovarian tissue preservation and egg donation should be discussed with the patient. The aim of the fertility preservation should be to offer the most efficient approach in accordance with the legal country-specific regulations, while not increasing the oncological risk [IV, B].
- Any pregnancy following fertility sparing therapy should be considered as a high-risk pregnancy. Following simple or radical trachelectomy with placement of a permanent cerclage, delivery can only be performed by cesarean section [IV, B].
- Although evidence is limited, several antenatal management tools can be considered following fertility sparing therapy including screening and treatment of asymptomatic bacteriuria, screening for cervical incompetence and progressive cervical shortening by transvaginal ultrasonography, fetal fibronectin testing, screening (and treatment) for asymptomatic vaginal infection, vaginal progesterone application, total cervical closure according to Saling and cervical cerclage, if not placed during trachelectomy [IV, C].
- Routine hysterectomy after completion of childbearing is not mandatory [V, D].

### Invasive Cervical Cancer Diagnosed on a Simple Hysterectomy Specimen

#### General Recommendations

- Management of disease found after SH should be based on expert pathology review and discussed in a multidisciplinary tumor board. In general, management of occult disease follows the principles of the standard management, and is based on pathologic findings, and clinical staging. Treatment strategy should aim to avoid combining further surgery and radiotherapy because of the high morbidity after combined treatment [III, B].
- Before making further management decisions, optimal imaging is necessary to evaluate the local and regional (nodal) disease status. Optimal imaging follows the same recommendations as that for the standard management [III, B].
- When surgical staging of nodal disease is indicated (see below for details), it can be considered either as an isolated (preferentially laparoscopic) procedure or as the first step of surgical management in radiologic node negative patients. Surgical staging of nodal disease can also be considered to assess inconclusive nodes at imaging. SLN biopsy cannot be performed in the absence of the uterus. Any suspicious LN should be sent for intraoperative assessment (frozen section) [III, B].
- Para-aortic LN dissection, at least up to inferior mesenteric artery, may be considered for staging purposes in patients with positive pelvic nodes at imaging, or at frozen section [IV, C].

#### Management of Patients with T1a1 and T1a2 Disease

- In patients with T1a1 tumor regardless of LVSI status and T1a2 tumor LVSI negative with clear margins in the hysterectomy specimen, no additional treatment is recommended [III, B].
- Surgical LN assessment can be considered in T1a1 tumors with LVSI and it should be performed in T1a2 LVSI positive cases [III, B].

#### Management of Patients with T1b1 Disease, with Clear Margins and Without Residual Tumor

- Surgical LN staging is recommended in patients with T1b1 tumor with clear margins and absence of residual tumor on imaging (including non-suspicious LN). In case of histological evidence of PLN involvement, definitive CTRT is recommended and PALND, at least up to inferior mesenteric artery, may be considered for staging purposes [III, B].
- In pathologically node negative patients with T1b1 disease, potential disease in the parametria should be addressed. Parametrectomy and upper vaginectomy should be considered [III, B].
- Radiotherapy can be considered as an alternative modality to surgical treatment, considering the risk-benefit of repeat surgery [IV, C].

#### Management of Patients with ≥ T1b2 Disease, Involved Surgical Margins and/or Residual Tumor (Including LN)

- For patients with free surgical margins and in the absence of residual tumor on imaging (including non-suspicious LN), (chemo)radiotherapy is recommended as a treatment that avoids further surgical management [IV, B].
- Radical surgery (pelvic lymphadenectomy, parametrectomy and resection of the upper vagina) is an option in selected patients without expected indication for adjuvant (chemo)radiotherapy. If surgery has been performed, indications for adjuvant (chemo)radiotherapy follow the general recommendations [IV, B].
- If there is residual tumor on imaging (including suspicious LN), or involved surgical margins, CTRT with or without BT is the treatment of choice (see principles of radiotherapy) [III, B]. Para-aortic LN dissection, at least up to inferior mesenteric artery, may be considered for staging purposes in patients with positive pelvic nodes and negative paraaortic LN on imaging [IV, C].

#### Management of Locally Advanced Cervical Cancer (T1b3-T4a)

- Definitive radiotherapy should include concomitant chemotherapy whenever possible [I, A].
- IGBT is an essential component of definitive radiotherapy and should not be replaced with an external boost (photon or proton). If BT is not available, patients should be referred to a center where this can be done [III, B].
- General recommendations for prescription of CTRT and IGBT are as follows (details given in the section on principles of radiotherapy) [III, B]:
  - 3D imaging (preferentially both MRI and (PET-CT) with the patient in the treatment position should be used for target contouring.
  - It is recommended to deliver external beam radiotherapy (EBRT) with a dose of 45 Gy/25 fractions or 46 Gy/23 fractions by use of intensity-modulated or volumetric arc technique.
  - Additional dose of radiation should be applied to pathological LN on imaging, preferentially using a simultaneous integrated boost (60 Gy EQD2, combined EBRT and estimated dose from IGBT).
  - Concomitant weekly cisplatin is standard. However, weekly carboplatin or hyperthermia can be considered as an alternative option for patients not suitable for cisplatin.
  - Image-guided adaptive brachytherapy (IGABT) (preferentially MRI) including access to intracavitary/interstitial techniques are needed to obtain a sufficiently high dose to ensure a high rate of local control in advanced cases with poor response to initial CTRT. This is especially important for non-squamous histology.
  - Boosting of the primary tumor and/or the parametria by use of EBRT should be avoided.
  - The overall treatment time including both CTRT and IGBT should aim to not exceed 7 weeks.
- PALND (at least up to inferior mesenteric artery) may be used to assess the need for elective para-aortic EBRT in patients with negative para-aortic lymph nodes (PALN) and positive PLN on imaging [IV, C].
- If PALND is not performed, risk assessment for microscopic para-aortic nodal involvement and the indication for elective para-aortic irradiation can be based on the number of level 1 positive nodes (external iliac, interiliac, internal iliac) on imaging (e.g. >2 positive nodes). However, elective para-aortic radiation should always be applied in patients who on imaging have even one positive node at level 2 (common iliac) and above. The groin should also be included in the elective target for patients with tumor involvement of the lower-third of the vagina [IV, B].
- Surgical removal of large pathological pelvic and/or para-aortic nodes before definitive CTRT is not routinely recommended [IV, D].
- NACT in patients who otherwise are candidates for upfront definitive CTRT and IGBT is not recommended outside of clinical trials [II, D].
- Adjuvant chemotherapy following definitive CTRT and IGBT does not improve survival and enhances toxicity and should not be used outside clinical trials [IV, D].
- Adjuvant/completion hysterectomy after definitive CTRT and IGBT should not be performed since it does not improve survival and is associated with both increased perioperative and late morbidities [II, E].
- Patients with a persistent tumor 3–6 months after definitive CTRT and BT and without evidence of regional or metastatic disease should be referred to specialized centers for evaluating the necessity and the possibility of performing salvage surgery (see management of recurrent disease and follow-up sections) [IV, B].

#### Role of Surgery in T1B3 and T2a2 (LN Negative) Tumors

- There is limited evidence to guide the choice between surgical treatment vs CTRT with IGBT in LN negative patients with T1b3 and T2a2 tumors. Histology, tumor size, completeness of the cervical rim, uterine corpus invasion, magnitude of vaginal invasion, age, comorbidity, menopausal status, body mass index, hemoglobin and experience with type C radical hysterectomy are some of the factors to consider [IV, B].
- For surgery, avoidance of the combination of radical surgery and post-operative external radiotherapy requires acceptance for modifications of the traditional selection criteria (tumor size, degree of invasion, LVSI) for adjuvant treatment [IV, B].
- The patient should be discussed in a multidisciplinary team and should be counseled for the advantages and disadvantages of both treatment options (surgery vs radiotherapy) in relation to the individual presence of prognostic factors [IV, A].
- Given the limited number of patients with T1b3 and T2a2 (<10%) tumors, referral to highly specialized centers for treatment is recommended [IV, A].
- Type C radical hysterectomy is recommended. LN staging should follow the same principles as in T1b1-2 tumors [IV, A].
- NACT followed by radical surgery should not be performed outside clinical trials [I, E].

### Recurrent/Metastatic Disease

#### General Recommendations

- Treatment of recurrent disease requires centralization and involvement of a broad multidisciplinary team including a gynecological oncologist, radiation oncologist, radiologist, pathologist, medical oncologist, urologist, and plastic surgeon. A structured program for multidisciplinary diagnostic work-up, treatment, and follow-up must be present in centers responsible for the treatment [IV, A].
- Participation in clinical trials is encouraged [V, B].
- Early involvement of a palliative care specialist is encouraged [V, B].
- The patient should be carefully counseled regarding treatment options, risks and consequences [V, A].

#### Diagnostic Work-up

- The aim of the diagnostic work-up is to determine the extent of the locoregional and/or metastatic disease [V, B].
- The recurrence should be confirmed by histological examination if feasible [IV, B].
- Patients with multiple nodal/distant metastases (ie, not oligometastatic disease) or multifocal local disease with extensive pelvic wall involvement should not be considered as candidates for radical treatment [IV, D].
- Patients with oligometastatic or oligorecurrent disease should be considered for radical and potentially curative treatment options [IV, B].
- The prognostic factors should be evaluated carefully and balanced in relation to the major morbidity caused by the treatment [IV, A].

#### Locoregional Recurrent Disease - Central Pelvic Recurrence After Primary Surgery

- Definitive CTRT combined with IGABT is the treatment of choice in radiotherapy naïve patients [IV, A]. The use of boost by external beam techniques to replace IGBT is not recommended [IV, D].
- Small superficial lesions (ie, <5 mm thickness) in the vagina may be treated by IGBT using a vaginal cylinder, ovoids, or mold, whereas other lesions usually require combined intracavitary-interstitial techniques [IV, C].

#### Locoregional Recurrent Disease - Pelvic Sidewall Recurrence After Primary Surgery

- Definitive CTRT is the preferred option in radiotherapy naïve patients [IV, A].
- When radical radiotherapy is not feasible, extended pelvic surgery can be considered. Surgery must aim for a complete tumor resection (R=0) also with the help of special techniques (laterally extended endopelvic resection (LEER), out of box procedures), if required [IV, B].
- Combined operative-radiotherapy procedures using intra-operative radiotherapy or IGBT are an option if free surgical margins are not achievable [IV, B].

#### Locoregional Recurrent Disease - Central Pelvic or Pelvic Sidewall Recurrence After Radiotherapy

- Pelvic exenteration is recommended for central pelvic recurrence where there is no involvement of the pelvic sidewall, extrapelvic nodes or peritoneal disease [IV, B].
- Reirradiation with IGABT for central recurrences could be considered in selected patients taking into account volume of the disease, or time from the primary radiotherapy and total dose administered initially. This must be performed only in specialized centers [IV, C].
- In patients with pelvic sidewall involvement, extended pelvic surgery can be considered in specialized centers. Surgery must aim for a complete tumor resection (R=0) also with the help of special techniqu**e**s (LEER, out of box procedures), if required [IV, B].
- Patients who are not candidates for extensive surgery should be treated with systemic chemotherapy. Additional treatment can be considered depending of the response [IV, B].

#### Oligometastatic Recurrences

- Localized para-aortic, mediastinal, and/or peri-clavicular recurrences out of previously irradiated fields may be treated by radical EBRT with or without chemotherapy [IV, C].
- The therapeutic effect of nodal resection/debulking is unclear and should, if possible, be followed by radiotherapy [IV, C].
- The management of “oligo” organ metastases (lung, liver, etc.) should be discussed in a multidisciplinary setting including the team involved in the treatment of the organ-affected metastasis. Treatment options are represented by local resection, thermal ablation, interventional BT, or stereotactic ablative radiotherapy according to the size and localization [IV, B].

#### Distant Recurrent and Metastatic Disease

- Patients with recurrent/metastatic disease should have a full clinical-diagnostic evaluation to assess the extent of disease and the most appropriate treatment modality including best supportive care [V, A].
- Platinum-based chemotherapy±bevacizumab is recommended for chemo-naïve, medically fit patients with recurrent/metastatic disease. Carboplatin/paclitaxel and cisplatin/paclitaxel are the preferred regimens [I, A].
- The addition of bevacizumab to platinum-based chemotherapy is recommended when the risk of significant gastrointestinal/genitourinary toxicities has been carefully assessed and discussed with the patient [I, A].
- The addition of pembrolizumab to platinum-based chemotherapy±bevacizumab is recommended in patients with PD-L1 positive tumors, assessed as combined positive score (CPS) of 1 or more [I, A].
- Patients who progressed after first-line platinum-based chemotherapy should be offered treatment with the anti PD-1 agent, cemiplimab, regardless of PDL-1 tumor status as long as they had not previously received immunotherapy [I, A].
- Patients with distant metastatic disease at diagnosis, who have responded to systemic chemotherapy, could be considered for additional radical pelvic radiotherapy (including IGBT in selected cases). Those with residual oligometastatic disease after systemic treatment could also be considered for additional regional treatment (surgery, thermal ablation, radiotherapy) to involved sites [IV, C].
- Inclusion of patients with recurrent/metastatic disease in clinical trials is strongly recommended [V, A].

### Follow-up During and After Treatment/Long-term Survivorship

#### General Recommendations

- Patients should be informed and educated at the time of diagnosis and throughout follow-up about signs/symptoms of recurrence. They should be informed about possible side effects (by physicians, nurses, brochures, videos, etc.) [V, A].
- A network of healthcare providers including all care providers should be involved in the care of survivors (eg, primary care physicians, gynecologists, psychologists, sexologists, physiotherapists, dieticians, social workers) for the follow-up [V, A].
- Follow-up strategy should be individualized in terms of intensity, duration and procedures, taking into account individual risk assessment [V, A]. Available prognostic models, such as the Annual Risk Recurrence Calculator available on the ESGO website can be used to tailor surveillance strategy in an individual patient [IV, B].
- Follow-up should be centralized/coordinated in a center specialized in the treatment and follow-up of gynecological cancer patients [IV, A].
- Follow-up is designed to monitor disease response, to detect recurrence and to screen for subsequent primary tumors [V, B].
- Regular and systematic monitoring of side effects and quality of life should be performed to improve the quality of care [V, A].
- Prevention and early detection of immediate and persistent symptoms and side effects of the different cancer treatments and the individual patient supportive care needs should be identified and established at diagnosis and monitored throughout the follow-up [V, A].
- All side effects should be identified and treated if possible, namely physical and psychosocial [V, A].
- The development of an individual survivorship monitoring and care plan is recommended [V, B].
- Recommendations for a healthy life style should include smoking cessation, regular exercise, healthy diet and weight management [V, B].
- Clinical trials should address long-term cancer survivorship and should include patient related outcomes [V, B].
- Quality control of care should be established [V, B].
- Each visit should be composed of the following [V, A]:
  - Patient history (including identification of relevant symptoms and side effects)
  - Physical examination (including a speculum and bimanual pelvic examination)
  - Imaging and laboratory tests should be performed only based on risk of recurrence, symptoms or findings suggestive of recurrence and/or side effects.
  - Regular review of an ongoing survivorship plan that can be shared with other healthcare providers.
- Oncological follow-up
  - Patients should be educated about symptoms and signs of potential recurrence [V, A].
  - Appropriate imaging test (MRI, ultrasound for pelvic assessment, CT scan or PET-CT for systemic assessment) should be used in symptomatic women [IV, A].
  - In case of suspected tumor persistence, recurrence or second primary cancer, histological verification is strongly recommended [V, A].
  - Vaginal vault cytology is not recommended [IV, D].
  - After fertility sparing treatment, follow-up should include HPV testing (at 6–12 and 24 months) [V, A].
- Monitoring of quality of life and side effects
  - Quality of life and side effects should be regularly assessed at least by the physicians/clinical care nurses and if possible by patients (using patient related outcomes). Patient self-reporting of side effects should be encouraged during and after treatment with the same frequency as medical visits [IV, B].
  - A checklist of potential main side effects should be included in the patient survivorship monitoring and care plan (eg, sexual dysfunction, lymphedema, menopausal symptoms and osteoporosis, genito-urinary and gastrointestinal disorders, chronic pain, fatigue) [IV, A].
  - After CTRT and BT, patients should be counseled about sexual rehabilitation measures including the use of vaginal dilators. Topical estrogens are indicated [IV, B].
  - Hormone replacement therapy is indicated to cervical cancer survivors with premature menopause and should be consistent with standard menopausal recommendation [IV, B]. Physical and lifestyle changes may also help [V, C].
  - Bone status should be assessed regularly in patients with early menopause [V, B].

#### Follow-up After Definitive CTRT and BT

- Follow-up should be performed/coordinated by a physician experienced with follow-up care after radiotherapy and BT including monitoring of early, and late treatment-related side effects [V, A].
- The same imaging method used at the start of treatment should be used to assess tumor response [V, B].
- Routine biopsy to assess complete remission should not be performed [IV, D].
- Cytology is not recommended in detecting disease recurrence after radiotherapy [IV, D].
- Imaging (pelvic MRI±CT scan or PET-CT) should be performed not earlier than 3 months after the end of treatment [IV, B].
- In patients with uncertain complete remission at 3 months post-radiotherapy, the assessment should be repeated after an additional 2–3 months with biopsy if indicated [IV, B].

### Quality of Life and Palliative Care

#### General Recommendations

- Early palliative care, integrated with oncological treatments, should be offered by the clinical team to all the patients diagnosed with advanced cervical cancer for managing symptoms and improving quality of life. A multidisciplinary approach must be included in the care plan with discussion and planning for specific treatment of these symptoms [IV, A].

#### Pain

- Opioids are the main analgesics for the treatment of moderate to severe cancer-related pain; the first option is oral morphine [I, A]; but other opioids and alternative routes (transdermic, subcutaneous) can be required in specific situations (ie, intestinal obstruction, problems with swallowing, renal failure) [III, B].
- If opioids alone do not provide sufficient pain relief cancer-related neuropathic pain should be treated with a combination of opioids and carefully dosed adjuvants (gabapentin, pregabalin, duloxetine, and tricyclic antidepressants) [III, B].
- Severe pelvic cancer pain unresponsive to an opioid regimen can benefit from other procedures like plexus block or spinal analgesia techniques [III, B].
- Palliative EBRT (if feasible) is effective for painful pelvic progression and bone metastasis [IV, B].

#### Renal Failure

- Urinary derivation by ureteral stent or percutaneous nephrostomy should be considered to treat renal failure caused by tumoral obstruction. There are no clear guidelines to predict which patients will benefit from these procedures in terms of survival and quality of life, and its indication should be discussed carefully [IV, C].

#### Malignant Intestinal Obstruction

- Medical management of malignant intestinal obstruction consists of antisecretory, corticosteroids, and antiemetic drugs. A nasogastric tube is recommended if vomiting and discomfort persist in spite of medical management. Surgical procedures can be considered in selected patients [IV, B].

#### Vaginal Bleeding and Discharges

- In the case of vaginal bleeding, vaginal packing, interventional radiology (selective embolization) or palliative radiotherapy (if feasible) are recommended. There is not enough evidence to prefer one over the other. In the case of massive refractory bleeding, palliative sedation can be considered. Malodorous vaginal discharge can be improved with vaginal washing and the use of a vaginal metronidazole tablet [IV, B].

#### Psychosocial Suffering

- In patients with cervical advanced cancer, a multidisciplinary approach of physicians, nurses, psychologists, social workers, and community health workers is needed to manage psychosocial and spiritual suffering associated with social stigma deriving from genital disease, malodorous vaginal discharge, etc [IV, A].

### Cervical Cancer in Pregnancy

#### General Recommendations

- Every patient diagnosed with cervical cancer in pregnancy must be counseled by a multidisciplinary team. This team should consist of experts in the fields of gynecological oncology, neonatology, obstetrics, pathology, anesthesiology, radiation oncology, medical oncology, psycho-oncology, and, spiritual and ethical counseling. National or international tumor board counseling may be considered [V, A].
- Given the large spectrum of therapeutic options, the multidisciplinary team should recommend a treatment plan according to the patient’s intention, tumor stage, and gestational age of pregnancy at the time of cancer diagnosis. The primary aims of the recommended treatment plan are the oncological safety of the pregnant woman as well as the fetal survival without additional morbidity [V, A].
- Treatment of patients with cervical cancer in pregnancy should be exclusively done in gynecological oncology centers associated with the highest level perinatal center with expertise in all aspects of oncologic therapy in pregnancy and intensive medical care of premature neonates [V, A].

#### Clinical and Imaging Diagnosis

- Clinical examination and histological verification of cervical cancer are mandatory [IV, A].
- Pathological confirmation may be obtained by colposcopy oriented biopsy or small cone (appropriate only during the first trimester of pregnancy, endocervical curettage is contraindicated) [IV, C].
- Preferred imaging modalities for clinical staging in patients with cervical cancer in pregnancy include pelvic MRI or expert ultrasound as part of the primary work-up. Gadolinium-based contrast agents should be avoided [III, A].
- The use of whole-body diffusion-weighted imaging MRI (WB-DWI/MRI) can reliably obviate the need for gadolinium contrast and radiation for nodal and distant staging during pregnancy. If not available, chest CT scan with abdominal shielding is an alternative. PET-CT should be avoided during pregnancy [IV, B].

#### Oncological Management

- Tumor involvement of suspicious nodes should be histologically confirmed because of its prognostic significance and the impact on the management up to 24 weeks of gestation (fetal viability) [IV, A].
- Minimally invasive approach could be considered before 14–16 weeks of gestation; however, the sentinel node biopsy concept using indocyanine green is still experimental [IV, C].
- Several treatment modalities are available and should be discussed with the patient taking into account the tumor stage, gestational week of pregnancy and the patient’s preferences [IV, B]:
  - Delay of oncological treatment until fetal maturity (if possible >34 weeks of gestation) and initiate cancer-specific treatment immediately after delivery by cesarean section. This option might be considered if the term or fetal maturity is approaching.
  - Conization or simple trachelectomy in order to completely remove the tumor, obtain free margins and perform nodal staging if needed, with the intention to preserve the pregnancy.
  - Radical surgery or definitive CTRT according to the disease stage as recommended outside pregnancy, if the woman decides not to preserve the pregnancy. Pregnancy termination is recommended before any treatment after the first trimester, and fetus evacuation before CTRT, if possible.
  - Chemotherapy until term of pregnancy (37 weeks of gestation) and initiation of definitive cancer-specific treatment immediately after delivery by cesarean section. At least a 2 week interval between chemotherapy and surgery is recommended. In patients with locally advanced disease or residual tumor after surgical procedure that cannot be completely removed (risk of premature rupture of amniotic membranes and/or cervical insufficiency), chemotherapy based on cisplatin or carboplatin can be considered starting after 14 weeks of pregnancy. Combination with taxanes is an option. Bevacizumab and checkpoint inhibitors are contraindicated.
  - Before starting each cycle of chemotherapy, an assessment of treatment response should be made by clinical examination and transvaginal or transrectal ultrasound. If no response is achieved after 2 cycles of chemotherapy during pregnancy, treatment strategy should be re-evaluated.

#### Pregnancy Management

- Spontaneous delivery appears to have negative prognostic impact in patients with cervical cancer in pregnancy. Thus, cesarean section is the recommended mode of delivery [IV, B].
- At the time of cesarean section, definitive cancer specific treatment should be performed corresponding to that of non-pregnant women, taking into account the treatment that has already been given during pregnancy [IV, A].

### Rare Tumors

- Histopathological diagnosis of rare cervical tumors needs confirmation (second opinion) by an expert pathologist [IV, A].
- Treatment and care of rare cervical tumors needs to be centralized at referral centers and discussed in a multidisciplinary tumor board [IV, A].

## Algorithms

### Management of T1a Disease

**Figure:**
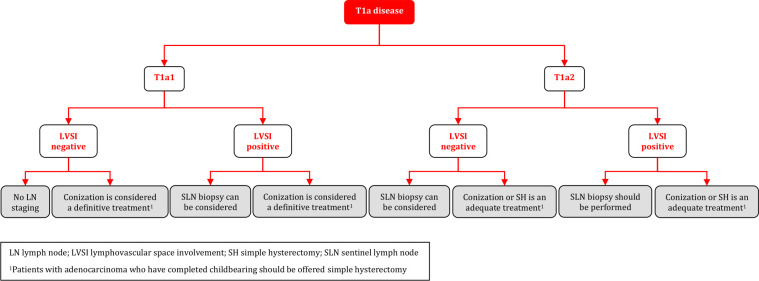


### Primary Treatment of T1b1, T1b2, and T2a1 Tumors

**Figure:**
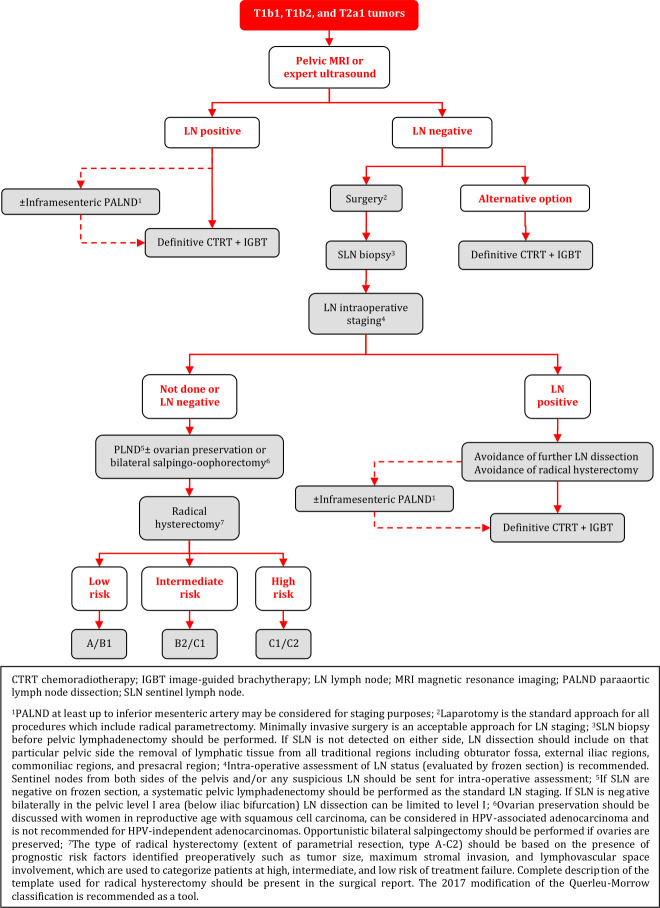


### Adjuvant Treatment of T1b1, T1b2, and T2a1 Tumors

**Figure:**
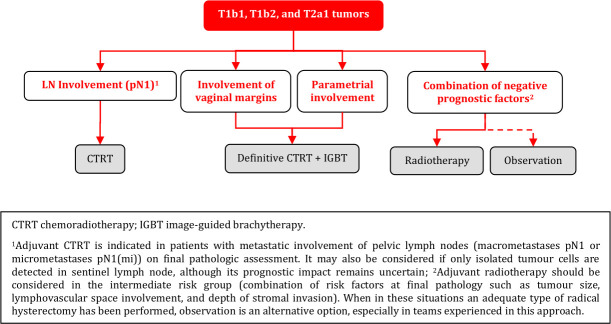


### Fertility Sparing Treatment - Selection of Candidates

**Figure:**
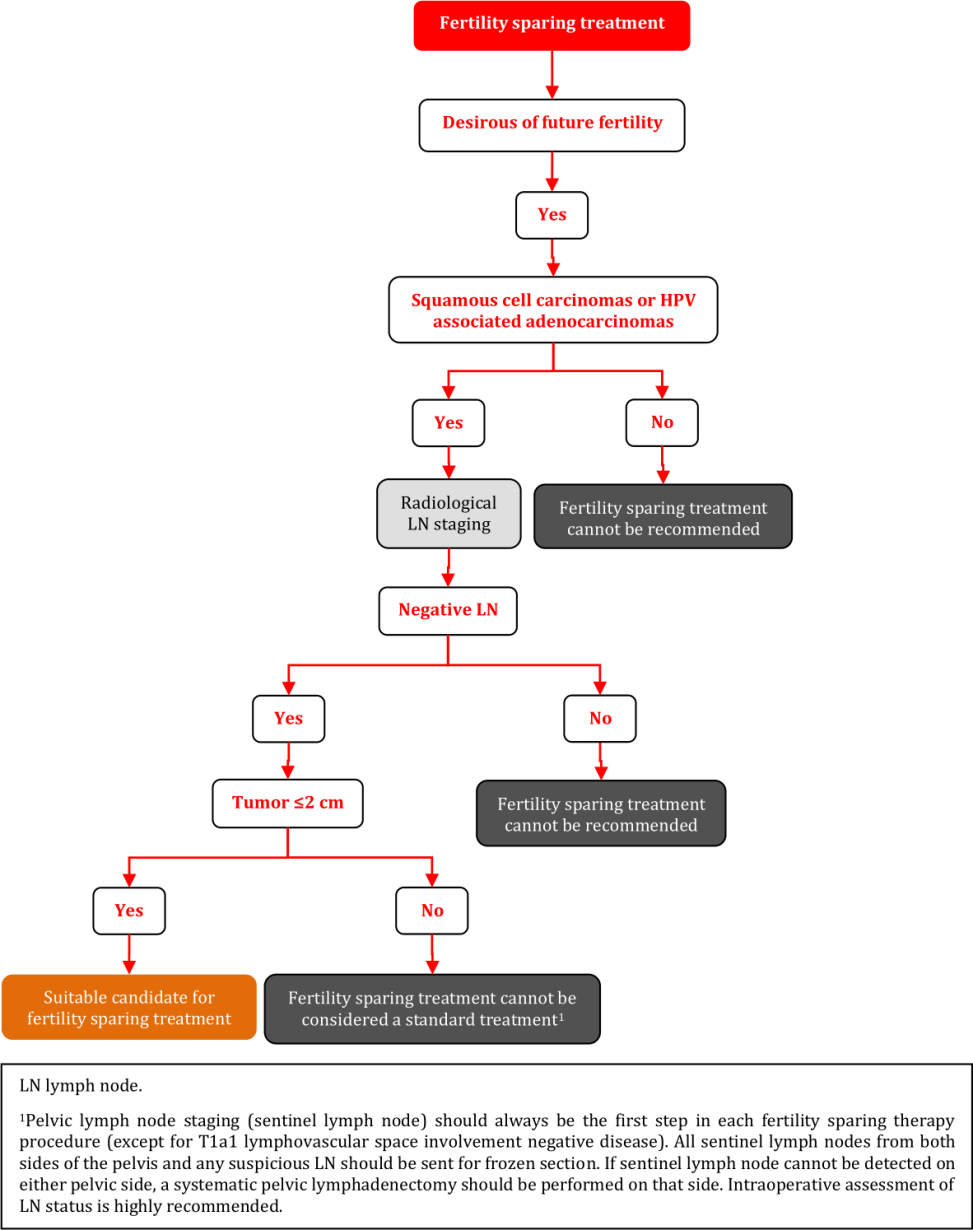


### Fertility Sparing Treatment - Management

**Figure:**
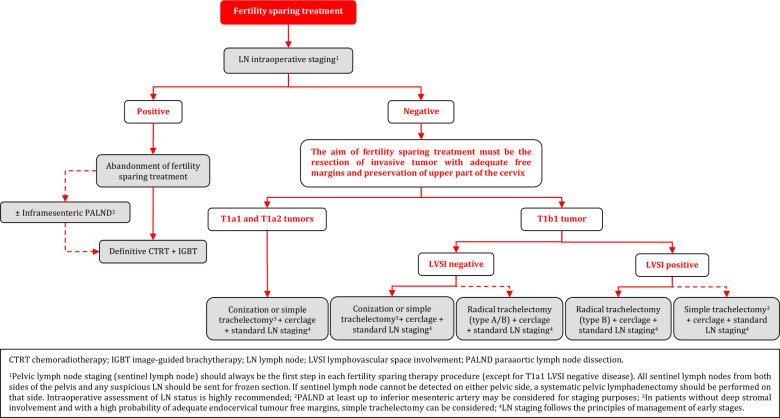


### Invasive Cervical Cancer Diagnosed on a Simple Hysterectomy Specimen

**Figure:**
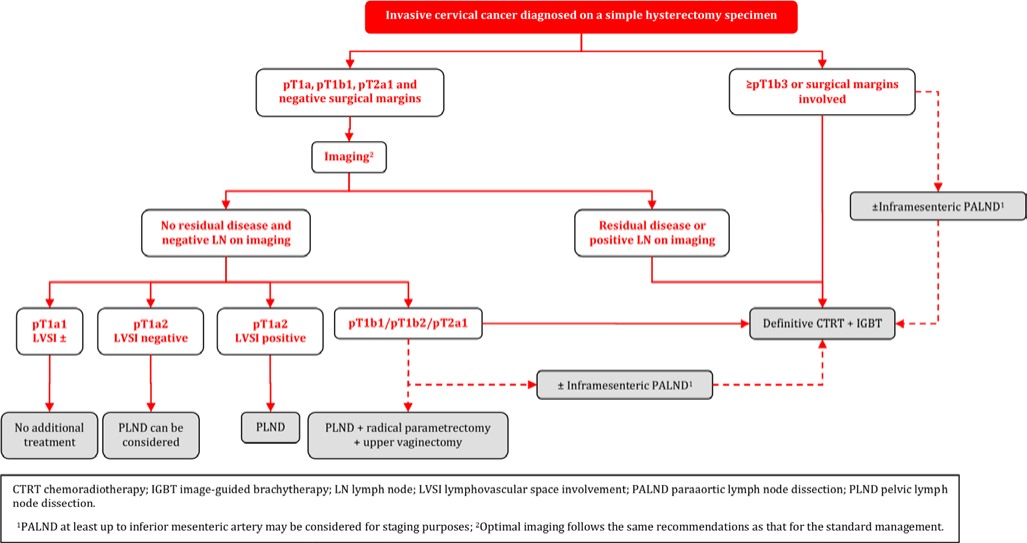


### Management of Locally Advanced Disease

**Figure:**
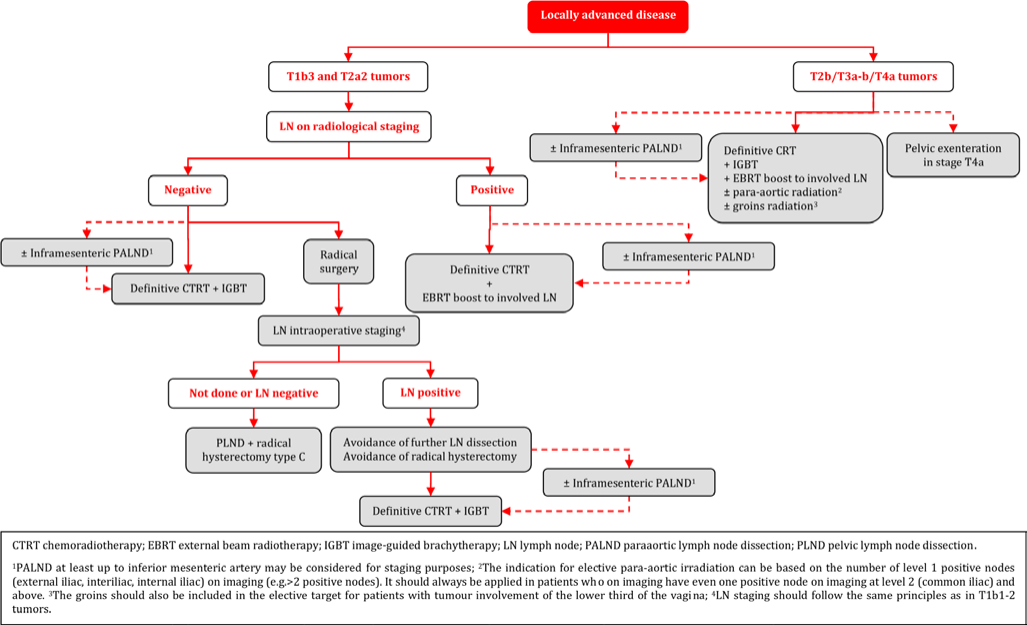


### Cervical Cancer in Pregnancy

**Figure:**
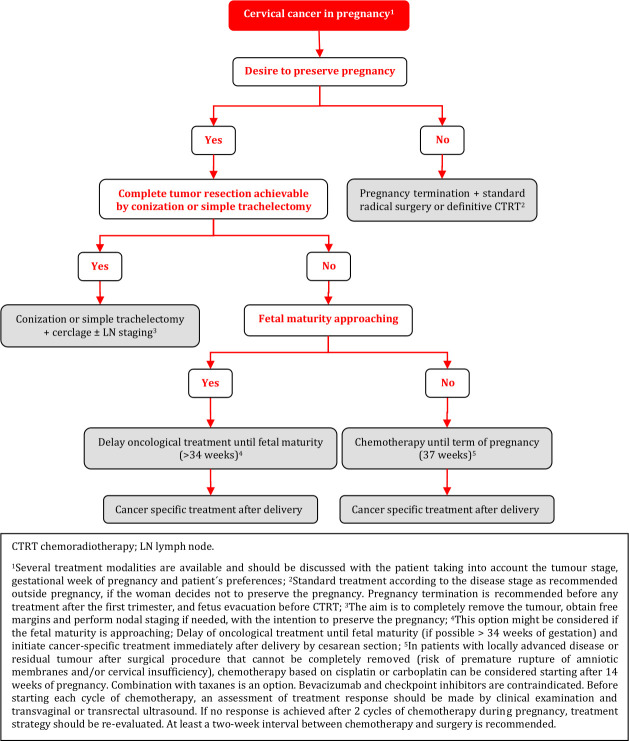


### Recurrent Disease

**Figure:**
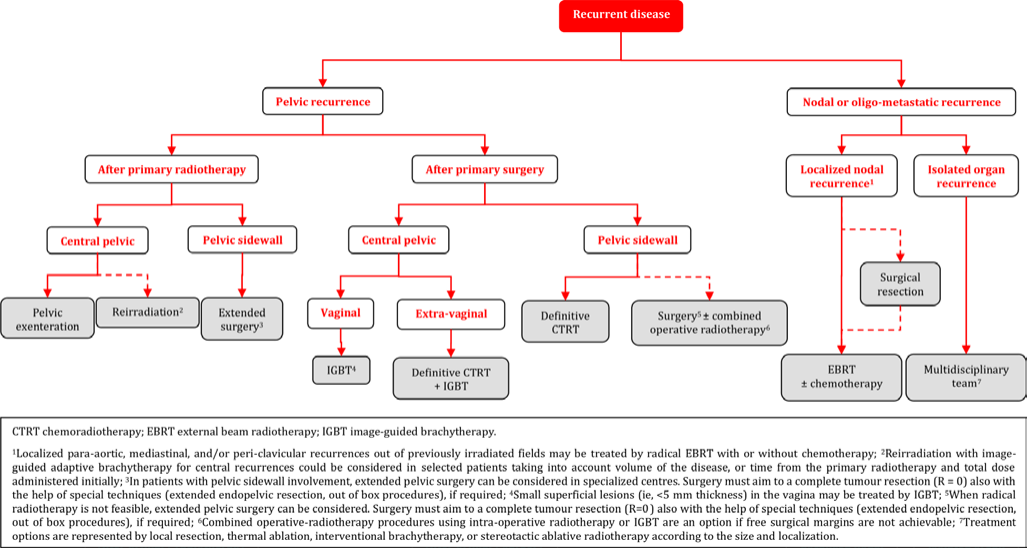


### Distant Recurrent and Metastatic Disease

**Figure:**
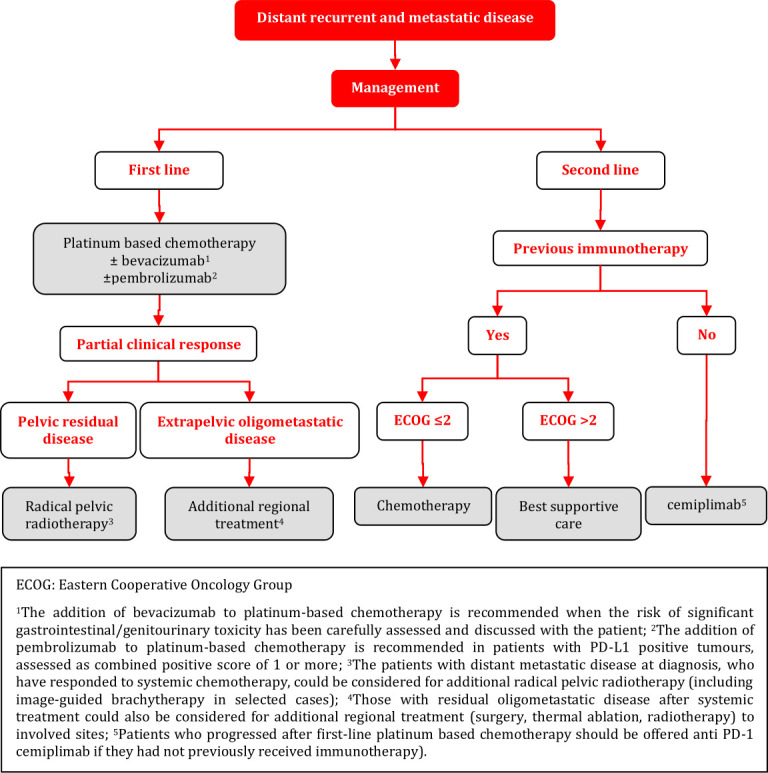


## Principles of Radiotherapy

### Definitive CTRT and BT - General Aspects

Definitive management (ie, without tumor related surgery) consists of EBRT with concomitant platinum-based chemotherapy and BT. Delay of treatment and/or treatment interruptions have to be prevented to avoid tumor progression and accelerated repopulation. The overall treatment time including both EBRT and BT should therefore not exceed 7 weeks.

### Definitive CTRT and BT

#### CTRT

Target contouring for EBRT should be based on 3D imaging (preferably fused MRI and PET-CT) performed in the supine treatment position. Controlled bladder filling is recommended to minimize uterus movements and to push the intestines away. The result of the gynecological examination (ie, clinical drawing and description) as well as diagnostic imaging should be available during the contouring phase. A contouring protocol including a margin strategy for handling of internal movement (ITV) should be used to minimize irradiation of organs at risk. The EMBRACE II protocol may serve as a template. The tumor related target volume for EBRT (CTV-T-LR) includes the primary cervical tumor (GTV-T), the uterus, parametria and upper vagina (or minimal 2 cm tumour-free margin below any vaginal infiltration respectively) and is optimally defined on MRI with assistance of the clinical findings.

The elective target (CTV-E) includes the obturator, internal, external and common iliac and presacral regions. The inguinal nodes should be included if the primary tumor involves the distal third of the vagina. A reduced elective target volume for EBRT without the common iliac nodes may be considered in low- and intermediate-risk T1b1 patients with negative LN and no LVSI. In case of PLN involvement indicating an increased risk of PALN spread (i.e.>2 pathological LN or involvement of common iliac region) and absence of surgical para-aortic staging, the elective target for EBRT should include the para-aortic region up to the renal vessels. In case of PALN involvement, the target volume includes at a minimum the region up to the renal vessels. Pathological macroscopic LN (GTV-N) are optimally localized with PET-CT and contoured on MRI.

The planning aim for EBRT is 45 Gy/25 fractions or 46 Gy/23 fractions using intensity-modulated radiotherapy/volumetric modulated arc therapy (IMRT/VMAT). A homogeneous dose from EBRT is needed in the central pelvis to ensure a safe platform for planning of BT. The use of an EBRT boost to the primary tumor and/or the parametria for complete or partial replacement of BT is not recommended.

Pathological macroscopic LN (GTV-N) should receive an EBRT boost. Simultaneous integrated boosting using coverage probability planning is recommended. Depending on nodal size and the expected dose contribution from BT a total dose of approximately 60 Gy EQD2 should be the aim of treatment. An alternative treatment option is surgical removal of enlarged nodes.

Image-guided radiotherapy with daily on-board 3D imaging is recommended for IMRT/VMAT to ensure safe dose application with limited PTV margins. Concomitant chemotherapy should be based on single-agent radiosensitizing chemotherapy, preferably cisplatin (weekly 40 mg/m²). If cisplatin is not applicable, alternative treatment options are weekly carboplatin (area under the curve (AUC) =2) or hyperthermia (if available). EBRT may also be applied without concomitant chemotherapy or hyperthermia according to patient selection (ie, patients unfit for any chemotherapy).

#### Brachytherapy

IGABT is recommended, preferably using MRI with applicator in place. Repeated gynaecologic examination is mandatory, and alternative imaging modalities such as CT scan and ultrasound may be used. The tumour-related targets for BT include: 1) the residual gross tumor volume (GTV-T_res_) after CTRT; 2) the adaptive high-risk clinical target volume (CTV-T_HR_) including the whole cervix and residual adjacent pathologic tissue; and 3) the intermediate-risk clinical target volume (CTV-T_IR_) taking the initial tumor extent into consideration. The BT applicator should consist of a uterine tandem and a vaginal component (ovoids/ring/mold/combined ring/ovoid). A combined intracavitary/interstitial implant is recommended in advanced cases to achieve the dose planning aim (see below), in particular in case of residual disease in the parametrium.

Ultrasound (transabdominal and/or transrectal) maybe used to intraoperatively support applicator insertion (avoidance of uterine perforation by the tandem, guidance of interstitial needles). In IGABT, the planning aim should be to deliver a BT dose of 40 to 45 Gy EQD2 to reach a total EBRT+BT dose of 85 to 95 Gy EQD2 (D90) (assuming 45 Gy through EBRT) to the CTV-T_HR_, equal to or greater than 60 Gy (D98) to the CTV-T_IR_, and equal to or greater than 90 Gy (D98) to the GTV-T_res_. The use of three dimensional and 2D dose volume and point constraints for rectum, bladder, vagina, sigmoid, and bowel are recommended, and they have to be based on the published clinical evidence. Even though point A dose reporting and prescription have been surpassed by the volumetric approach, a point A dose standard plan should be used as a starting point for stepwise treatment plan optimization to retain the pear shaped iso-dose pattern with a high central dose. This is especially important for the combined intracavitary/interstitial technique to avoid overloading of the interstitial needles.

BT should be delivered in several fractions as high dose rate (usually 3–4) with at least 6–8 hours interval or pulse dose rate delivered in one fraction (50–60 hourly pulses) or 2–3 fractions (15–24 hourly pulses) to respect the limitations of current radiobiological models for speed and capacity of radiation damage repair. In large tumors, BT should be delivered within 1 to 2 weeks toward the end of or after CTRT. In limited-size tumors, BT may start earlier during CTRT. For the tumour-related targets (GTV-T_res_, CTV-T_HR_, CTV-T_IR_), the use of external beam therapy for giving an extra dose (eg, parametrial boost, cervix boost) is not recommended, even when using advanced EBRT technology such as stereotactic radiotherapy or particle therapy. The use of a midline block for boosting the parametrium is not recommended when applying advanced image-guided radiotherapy and IGABT. Care should be taken to optimize patient comfort during (fractionated) BT. Preferably this includes a multidisciplinary approach. Intracavitary and combined intracavitary/interstitial BT implants should be performed under anesthesia.

### Adjuvant Radiotherapy or CTRT

Adjuvant radiotherapy or CTRT follows analog principles for target contouring, dose and fractionation as outlined for definitive treatment. Different concomitant and/or sequential chemotherapy schedules have been established including cisplatin alone or combinations of cisplatin with other agents such as 5-FU or paclitaxel. Carboplatin should be considered for patients unfit for cisplatin. The application of IMRT/VMAT and image-guided radiotherapy is recommended as treatment-related morbidity is reduced. Additional BT as part of adjuvant radiotherapy or CTRT should be considered only if a well-defined limited area accessible through a BT technique is at high risk of local recurrence (eg, positive resection margins in vagina or parametrium). Such adjuvant BT should follow the major principles outlined above for IGBT.

### Definitive 3D Conformal EBRT or CTRT and Radiography-based BT

Three-dimensional conformal radiotherapy alone or as definitive concomitant CTRT (platinum based) ± para-aortic radiotherapy and/or 2D radiography based BT is recommended, if intensity modulated radiotherapy and/or IGABT are not available. In case of 3D conformal radiotherapy and/or radiography based BT, the recommendations for EBRT and IGABT as outlined above in regard to target, dose, fractionation, and overall treatment time have to be respected as much as possible. A sequential LN boost is applied as appropriate after completion of 3D EBRT. Planning aim for BT should be based on point A. Dose to point A should be equal to or greater than 75 Gy (EQD2) in limited width adaptive CTV-T_HR_ (≤3 cm) and should aim at higher doses in large width adaptive CTV-T_HR_ (>4 cm). In addition, dose for the maximum width of the adaptive CTV-T_HR_ should be reported. Radiography based dose point constraints - plus 3D dose volume constraints as available - for rectum, bladder, vagina, sigmoid, and bowel are recommended, and must be based on published clinical evidence.

## Principles of Pathological Evaluation

### Requirements for Specimen Submitted for Pathological Evaluation

Patient information, previous cervical cytology, histological specimens, clinical and radiological data, colposcopic findings and information on previous treatment (eg, surgery, radiotherapy) need to be included on the specimen request form. Details of cytology, biopsy, and surgical specimen (cone/loop specimen, trachelectomy, type of hysterectomy, presence of ovaries and fallopian tubes, presence of LN and designation of the LN sites, presence of vaginal cuff, and presence of parametria) need to be itemized in the specimen request form. Biopsies and surgical specimens should be sent to the pathology department in a container with liquid fixative (‘‘clamping’’ of surgical specimens on a surface may be useful). If the local situation requires biobanking of fresh tissue, surgical specimens should be submitted fresh with minimum ischemia time. Cytology specimens should be sent to the pathology department preferentially as liquid-based cytology. Smear preparations are not recommended. The former is necessary when an HPV test is requested. Immunocytochemistry is possible on LBC but of limited extent (eg, CPS score for PD-L1 cannot be assessed). Cone/loop specimen should ideally be sent intact with a suture to identify the 12-o’clock position.

### Specimen Grossing and Sampling

#### Biopsy/Cone/Loop

Small biopsy specimens should be enumerated. The cone/loop specimens should be measured in three dimensions according to the recent ESGO/ESP recommendations. If the cone can be oriented properly, the anterior and the posterior half should be inked with separate colors. It should further be recorded if the specimen is complete or fragmented. If more than one piece of tissue is received, every piece should be measured in three dimensions. All specimens should be entirely submitted for microscopic examination. Inking of the surgical margins of cone/loop specimens is recommended. Dissection of cone/loop specimens should be performed in a standardized procedure. All the pieces submitted should be in consecutive numerical order. This is important because if tumor is present in more than one piece, it needs to be known whether these pieces are consecutive and, thus, a single tumor is present or whether the tumor is multifocal. It is recommended to place only one piece of tissue in each cassette. There are also techniques that allow embedding of more than one piece in a cassette if they are small enough. In cases that do not comprise intact cone/loops, serial radial sectioning and placing of each slice of tissue in a single cassette should be performed.

#### Trachelectomy

The upper (proximal) surgical margin of a trachelectomy specimen should be inked. The upper margin of a trachelectomy specimen should be sampled in its entirety in a way that allows to measure the distance of the tumor to the margin. The vaginal margin should also be inked and examined totally as radial sections if no tumor is seen grossly.

#### Hysterectomy

The description of the specimen (hysterectomy, trachelectomy, presence of ovaries and fallopian tubes, presence of LN and indication of the LN sites, presence of vaginal cuff and presence of parametria) should be recorded and checked for consistency with the description given in the specimen request form. The presence of any gross abnormality in any organ should be documented. The dimensions of the uterus for a hysterectomy specimen and the cervix for a trachelectomy specimen should be documented. The minimum and maximal length of the vaginal cuff should be documented. The size of the parametria should be documented in two dimensions (vertical and horizontal). Gross tumor involvement of the parametrium, vagina, uterine corpus, or other organs should be documented. The relationship of the cervical tumor to the vaginal and parametrial margins (and upper margin in case of a trachelectomy specimen) should be measured and appropriate sections taken to demonstrate this. Radial/circumferential and vaginal margins should be inked. The gross appearance of the cervix should be documented and any gross tumor mass measured. If visible, the site of a previous cone biopsy should be documented. Gross tumors should be measured in three dimensions, namely, the horizontal extent and the depth of invasion.

The tumor site within the cervix should be documented. The cervical tumor should be sampled to demonstrate the maximum depth of invasion, the relationship of the tumor with the surgical borders, and the extension to other organs. When the tumor is small (or with tumors that cannot be identified macroscopically), the cervix should be separated from the corpus, opened and processed as for a cone/ loop specimen. In the case of a large tumor, the hysterectomy or trachelectomy specimen should be opened in the sagittal plane. At least one block per centimeter of the greatest tumor dimension should be taken for large tumors.

Additional blocks including the cervix adjacent to the tumor should be taken to identify precursor lesions. The whole cervix should be sampled in the case of a small tumor or where no macroscopic tumor is identified. The uterine corpus, vagina, and adnexa should be sampled according to standard protocols if not involved by tumor. If the uterine corpus and/or adnexa are grossly involved, additional blocks should be sampled. The entire vaginal margin should be blocked. The parametria should be submitted totally for histological examination to assess tumor invasion and surgical margins. The use of large sections is optional and provides good information on tumor size and marginal status.

#### Lymph Nodes

All the LN should be submitted for histological examination. If the LN are grossly involved, representative samples are sufficient. If grossly uninvolved, each node should be sliced at 2 mm interval (eg, perpendicular to its longitudinal axis) and totally embedded. From each block, hematoxylin-eosin (H&E) sections should be taken. LN should be submitted in separate cassettes according to the site recorded on the specimen request form.

### Pathological Analysis of SLN

Intraoperative assessment of sentinel nodes is a reliable procedure but may miss micrometastases and isolated tumor cells. Intraoperative assessment should be performed on a grossly suspicious sentinel node and may be performed on a “non-suspicious” SLN because the confirmation of tumor involvement will result in abandoning a hysterectomy or trachelectomy. For intraoperative evaluation, the SLN should be sent to the pathology department in a container without liquid fixative. Intraoperative analysis requires gross dissection of the resected adipose tissue by the pathologist and selection of LN. It is important to leave some peri-nodal tissue allowing proper diagnosis of extra-nodal tumor spread. For a LN with obvious gross tumor, a single section is adequate for frozen section.

Frozen section may be combined with imprint cytology. The use of one step nucleic acid amplification is not recommended particularly due to the interference with benign epithelial inclusions in PLN. Any nonsuspicious sentinel node should be bisected (if small) or sliced at (approximately) 2 mm thickness and entirely frozen. From each sample, histological sections should be cut and stained by H&E. After frozen section analysis, the tissue should be put into a cassette, fixed in liquid fixative (preferably 4% buffered formalin) and subsequently processed and embedded in paraffin. If no metastases are present in the first section, SLN should undergo ultrastaging in definitive paraffin sections, including immunohistochemistry. A minimum procedure should include five serial sections at 200 µm. At least, at two levels an additional section must be cut and stained with a broad-spectrum cytokeratin antibody (eg, AE1/AE3). If the resources of the pathology lab allow, it is recommended to cut serial sections through the whole block (eg, at 100–200 µm) and to perform about additional cytokeratin immunostainings. Cytokeratin-positive cells should always be correlated with the morphology. Müllerian inclusions (endosalpingiosis, endometriosis) and mesothelial cells may rarely be present in pelvic and PALN and are cytokeratin positive.

### Requirements for Pathology Report

- Previous pertinent histological exams of the cervical lesion/cancer, even if diagnosed in another institution, should be revised and integrated in the final report (eg, cone biopsy and hysterectomy specimen)
- Description of the specimen(s) submitted for histological evaluation.
- Macroscopic description of specimen(s) (biopsy, loop/cone, trachelectomy, hysterectomy) including specimen dimensions (three dimensions), number of tissue pieces for loop/cones, and maximum and minimum length of vaginal cuff and the parametria in two dimensions.
- Macroscopic tumor site(s), if the tumor is grossly visible, in trachelectomy and hysterectomy specimens.
- Tumor dimensions should be based on a correlation of the gross and histological features and include the depth of invasion or thickness and the horizontal dimensions. Multifocal carcinomas are separated by uninvolved cervical tissue, each should be described and measured separately, and the largest used for tumor staging. In some studies, a distance of more than 2 mm was arbitrarily used to define multifocality. Multifocal carcinomas should not be confused with the scenario in which tongues or buds of invasive carcinoma originate from more than one place in a single zone of transformed epithelium
- Specimens from prior conization and subsequent conization, trachelectomy, or hysterectomy should be correlated for estimation of the tumor size. This is important since different specimens may have been reported at different institutions. It should also be recognized that simply adding the maximum tumor size in separate specimens may significantly overestimate the maximum tumor dimension. Histological tumor type according to the most recent WHO classification (currently 5th edition, 2020, in its updated version).
- Histological tumor grade if required. It needs to be stressed that currently grading remains of uncertain value for squamous cell carcinoma and most subtypes of adenocarcinoma. For adenocarcinoma, the growth pattern (Silva Classification) is recommended.
- The presence or absence of lymphatic vessel invasion (LVI), which may be confirmed by immunohistochemistry. The quantification of the number of lymph vascular vessels involved by tumor cells is not mandatory but advisable for future prospective studies.
- The presence or absence of venous invasion (V1) and of perineural invasion (Pn1).
- Coexisting precursor lesions such as squamous intraepithelial lesion/cervical intraepithelial neoplasia, adenocarcinoma in situ, stratified mucin-producing intraepithelial lesion and other pathological changes of the cervix.
- Measurements of tumor distance to all surgical margins (including minimum distance of uninvolved cervical stroma).
- Margin status (invasive and preinvasive diseases). Specify all the margin(s).
- LN status including SLN status, the total number of nodes found, the number of positive LN, the size of the largest metastatic focus, and the presence of extra-nodal extension. In the eighth UICC TNM edition isolated tumor cell deposits are no greater than 0.2 mm (200 µm) and should be reported as pN0 (i+). Micrometastasis (200 µm to 2 mm in diameter) are reported as pN1(mi).
- Pathologically confirmed (if required, including immunohistochemistry/HPV DNA) distant metastases.
- Provisional pathological staging pretumor board/multidisciplinary team meeting (UICC TNM 9th edition; American Joint Committee on Cancer, 9th edition).

### Items to be Included in the Pathology Report of Carcinomas of the Cervix

**Table:** 

Clinical/ Surgical	Macroscopic	Microscopic
Specimen(s) submitted	Specimen dimensions Loops/cones: Number of tissue pieces Transverse and anteroposterior diameters of ectocervix; Length Trachelectomy or Radical Hysterectomy: Weight and size Length of the cervix Vaginal cuff: Minimum and maximum length. Size of parametria (vertical and horizontal) Tumor size in three dimensions Macroscopic tumor site(s) LN: number and size	Tumor dimensions Horizontal extent (two measurements) Depth of invasion or thickness Histological tumor type LVSI Coexisting pathological findings Squamous intraepithelial lesion/cervical intraepithelial neoplasia (SIL/CIN). Adenocarcinoma in situ (AIS). Stratified mucin-producing intraepithelial lesion (SMILE). Tumor distance to all margins (proximal (if present) /radial/distal Margins status (invasive and preinvasive diseases). Specify the margin(s) LN status (SLN status, number involved/number retrieved, size of the largest metastatic focus, and presence of extra-nodal extension) Pathologically confirmed distant metastases Pathological staging (TNM category)
*Tumor dimension should be based on a correlation of the gross and histological features.		

### Ancillary Studies

All invasive carcinomas and adenocarcinoma in situ require an ancillary test to show the association with HPV. The most widely available and used technique is p16 immunohistochemistry (robust surrogate marker). Alternatively, HPV DNA or mRNA E6-E7 genes, can be detected by in situ hybridization or PCR-based techniques. HPV testing of cytological specimens requires liquid based cytology and uses mostly DNA-based or less frequently RNA-based molecular techniques. PD-L1 testing for the selection of immune checkpoint therapy is performed on tumor tissue, either biopsies or surgical specimens. PD-L1 expression seems to be frequently expressed in cervical carcinomas with special emphasis on locally advanced and HPV independent tumors. Standardized testing and evaluation including regular quality assessment is required to obtain a reliable patient selection for therapy. Prospective clinical trials will provide further information on the proper use of antibodies, assays and scoring systems. Further reading is available in Online Supplemental File 1

## Data Availability

All data relevant to the study are included in the article or uploaded as supplementary information.
