# Supplementary material for: ESGO/ESTRO/ESP Guidelines for the management of patients with cervical cancer – Update 2023*
Source: Int J Gynecol Cancer. 2023 Apr 26;33(5):649–66. doi: 10.1136/ijgc-2023-004429 (PMC10176411; doi:10.1136/ijgc-2023-004429)
Supplement: Supplementary data [file ijgc-2023-004429supp002.pdf]

APPENDIX 1. IDENTIFICATION OF SCIENTIFIC EVIDENCE

Literature search in MEDLINE

Research period	2017/01/01 - 2022/03/01
Indexing terms	5-fluorouracil, abdominal trachelectomy, abdominal radical trachelectomy, adenocarcinoma, adenoid basal carcinoma, adenoid basal cervical carcinoma, adenoid cystic carcinoma, adenoid cystic cervical carcinoma, adenosarcoma, adenosquamous carcinoma, advanced cervical cancer, advanced cervical carcinoma, advanced cervical disease, advanced disease, advanced stage, atypical carcinoid cervical tumour, atypical carcinoid tumour, basaloid cystic carcinoma, basaloid cystic cervical carcinoma, bevacizumab, biomarker, biopsy, bladder involvement, bleomycin, brachytherapy, brachytherapy boost, cancer antigen 125, cancer antigen 15-3, carboplatin, carcinoembryonic antigen, carcinosarcoma, cemiplimab, cervical adenoid-basal carcinoma, cervical adenoid cystic carcinoma, cervical atypical carcinoid tumour, cervical cancer, cervical carcinosarcoma, cervical carcinoma, cervical clear cell carcinoma, cervical high-grade neuroendocrine carcinoma, cervical low grade neuroendocrine tumour, cervical mixed large cell neuroendocrine carcinoma, cervical mucinous carcinoma, cervical mucinous tumour, cervical pure small cell carcinoma, cervical sarcoma, cervical sarcomatous tumour, cervical small cell neuroendocrine carcinoma, cervical stromal invasion, cervical stromal involvement, cervical typical carcinoid tumour, cervix cancer, cervix uteri, chemotherapy, circulating immune complexes, cisplatin, cis-diamminedichloroplatinum, cis-platinum, clear cell carcinoma, clear cell type, clear margin, clinical staging, clinical trial, clinically occult carcinoma, cold knife conization, colposcopy, combined large cell neuroendocrine carcinoma, combined positive score, combined small cell neuroendocrine carcinoma, complications, computed tomography, cone biopsy, cone resection, cone resection margins, conization, cryopreservation, cystoscopy, cytokeratin fragment 21-1, cytology, definitive treatment, diagnostic work-up, destructive techniques, early cervical cancer, early cervical carcinoma, early cervical disease, early disease, early stage,endovaginal ultrasound, endometrioid adenocarcinoma,excision, excisional techniques, external beam radiation therapy, external beam radiotherapy, extracervical tumour extension, fertility, fertility outcome, fertility preservation, fertility sparing, fertility sparing management, fertility sparing surgery, fertility sparing treatment, FIGO, FIGO staging system, follicular dendritic cell sarcoma, follow-up, follow-up procedures, follow-up protocols, frozen sections, germ cell tumour, gestation, high-grade neuroendocrine carcinoma, high sensitivity C-reactive protein, human papillomavirus-independent adenocarcinoma,human papillomavirus testing, hysterectomy, hysterectomy specimen, image guided adaptive brachytherapy, image guided radiotherapy, imaging, imaging modalities, imaging procedure,imaging test, immunosuppressive acidic protein, intensity modulated radiotherapy, intensive care, intensive care unit, interleukin 6, invasive cervical cancer, invasive cervical carcinoma, invasive cervical disease, invasive disease, invasive stage, isolated tumour cell, laparoscopic staging, laparoscopy, laparotomy, large cell neuroendocrine carcinoma, large loop excision of the transformation zone, laser ablation, laser ablation-destruction, laser conization, laser conization-excision, laser destruction, length of stay, leiomyosarcoma, local clinical diagnostic work-up, local radiological diagnostic work-up, locally advanced cervical cancer, locally advanced cervical carcinoma, locally advanced cervical disease, locally advanced disease, locally advanced stage, long-term survivorship, loop conization, loop electrosurgical excision procedure, low grade neuroendocrine tumour, lymphadenectomy, lymph node, lymph node assessment, lymph node dissection, lymph node staging, lymphovascular space involvement, macrometastatis, magnetic resonance imaging, malignant intestinal obstruction, malignant lymphoma, margin status, mature teratoma,mesonephric type, metastatic cervical cancer, metastatic cervical carcinoma, metastatic cervical disease, metastatic disease, microinvasive cancer, microinvasive cervical cancer, micrometastatis, mixed large cell neuroendocrine carcinoma, mortality rate, mortality analysis, mucinous carcinoma, mucinous tumour, mucoepidermoid carcinoma,multidisciplinary board, multidisciplinary setting, multidisciplinary team, multivariate analysis, myeloid sarcoma, neoadjuvant chemotherapy, neoadjuvant treatment, neonatal intensive care unit admission, nerve-sparing radical surgery, nerve-sparing robotic radical hysterectomy, nodal involvement, non-gestational choriocarcinoma, obstetric outcomes, obstetric risk, occult carcinoma, occult invasive carcinoma, occult invasive cervical cancer, oncologic outcome, oncologic risk, ovarian preservation, ovarian transplantation, ovarian transposition, oxaliplatin, paclitaxel, pain, palliative care, palliative chemotherapy, palliative management, palliative radiotherapy, palliative setting, palliative surgery, palliative systemic treatment, palliative treatment, paraaortic lymphadenectomy, paraaortic lymph node assessement, paraaortice lymph node dissection, parametrial resection, pathological analysis, pathological evaluation, pathological staging, pathology, pathology report, pathology report adequacy, patient-reported outcome, pelvic examination, pelvic lymph node assessement, pelvic lymph node dissection, pelvic lymphadenectomy, perioperative care, physical examination, platinum, platinum-based chemotherapy, positron emission tomography, positron emission tomography/computed tomography, postoperative care, postoperative complications, postoperative recurrence, pregnancy, preterm spontaneous rupture of membranes, preoperative brachytherapy, pregnancy, pregnancy outcome, pregnancy rate, pregnant patient, preoperative care, preoperative work-up, prognosis, prognostic factor, psychosocial suffering, pure small cell carcinoma, quality of health care, quality of life, radiation therapy, radical abdominal trachelectomy, radical surgery, radical trachelectomy, radical vaginal trachelectomy, radiochemotherapy, radiological staging, radiotherapy, rare tumour, rare cervical cancer, rare cervical carcinoma, rectal involvement, rectoscopy, recurrence, recurrent cervical cancer, recurrent cervical carcinoma, recurrent cervical disease, recurrent disease, reoperation, reproduction, reproductive techniques, residual disease, residual tumour, restaging, rhabdomyosarcoma, risk factors, robotic radical hysterectomy, sampling, sarcoma, sarcomatous tumour, sensitivity, sentinel lymph node, sentinel lymph node dissection, sentinel lymph node procedure, sentinel node, serum biomarker, serum marker, simple hysterectomy, simple hysterectomy specimen, specificity, simple trachelectomy, small cell neuroendocrine carcinoma, specialized center,specimen grossing, squamous cell carcinoma antigen, staging, staging procedures,stromal invasion, stromal involvement, supportive care,supportive management, supportive setting, supportive treatment, surgery, surgical lymph node assessment, surgical management, surgical margin, surgical outcome, surgical outcome criteria, surgical procedures, surgical resection, surgical staging, surveillance, survival rate, survival analysis, survivorship, terminal illness, terminally ill patient, tissue polypeptide antigen, TNM, TNM classification, total laparoscopic radical trachelectomy, trachelectomy, transplantation, transposition, transrectal ultrasound, treatment outcome, tumour-associated trypsin inhibitor, tumour necrosis factor alpha, typical carcinoid tumour, undifferentiated carcinoma,ultrastaging, ultrasound, upstaging, uterine cervix cancer, uterine transplantation, vaginal radical trachelectomy, vaginal trachelectomy, vascular space involvement, vascular endothelial growth factor, vincristine, yolk sac tumour.
Language	English
Study design	Priority was given to high-quality systematic reviews and meta-analyses but lower levels of evidence were also evaluated. The search strategy excluded editorials, letters, case reports and *in vitro* studies

Cibula D, *et al. Int J Gynecol Cancer* 2023; 33:649–666. doi: 10.1136/ijgc-2023-004429

APPENDIX 2. LIST OF THE 155 EXTERNAL REVIEWERS

Jafaru Abu, gynaecological oncologist (United Kingdom); **Jasimu Umar Adoke**, pathologist (Nigeria); **Hoda Al-booz**, clinical oncologist (United Kingdom); **Giovanni Aletti**, gynaecological oncologist (Italy); **Roberto Altamirano**, gynaecological oncologist (Chile); **Igor Aluloski**, gynaecological oncologist (Republic of North Macedonia); **Frédéric Amant**, gynaecological oncologist (Belgium); **Beatrice Anghel**, radiation oncologist (Romania); **Maarit Anttila**, gynaecological oncologist (Finland); **Ali Ayhan**, gynaecological oncologist (Turkey); **Paloma Badía Agustí**, gynaecological oncologist (Spain); **Elena Bakhidze**, gynaecological oncologist (Russia); **Joost Bart**, pathologist (Netherlands); **Anne-Sophie Bats**, gynaecological oncologist (France); **Mario Beiner**, gynaecological oncologist (Israel); **Virginia Benito**, gynaecological oncologist (Spain); **Kamil Biringer**, obstetrician gynaecologist (Slovakia); **Mazen Bishtawi**, gynaecological oncologist (Qatar); **Nicolò Bizzarri**, gynaecological oncologist (Italy); **Tatjana Bozanovic**, gynaecological oncologist (Serbia); **Kjersti Bruheim**, clinical oncologist (Norway); **Ewa Burchardt**, radiation oncologist (Poland); **Marta Caretto**, gynaecological oncologist (Italy); **Supriya Chopra**, radiation oncologist (India); **Nicoletta Colombo**, gynaecological oncologist (Italy); **Nicole Concin**, gynaecological oncologist (Austria); **Abel Cordoba**, radiation oncologist (France); **Sofia Córdoba Largo**, radiation oncologist (Spain); **Stefanie Corradini**, radiation oncologist (Germany); **Sabrina Croce**, pathologist (France); **Branko Cvjetićanin**, gynaecologist (Slovenia); **Alessandro D'Amuri**, pathologist (Italy); **Ademi Dafina**, clinical oncologist (Kosovo); **Kreshnike Dedushi-Hoti**, radiologist (Kosovo); **Anne De Middelaer**, patient (Belgium); **Vitaliana De Sanctis**, radiation oncologist (Italy); **Kalyan Dhar**, gynaecological oncologist (United Kingdom); **Antonino Ditto**, gynaecological oncologist (Italy); **Beth Erickson**, radiation oncologist (United States of America); **Brynhildur Eyjolfsdottir**, gynaecological oncologist (Norway); **Anna Fagotti**, gynaecological oncologist (Italy); **Hemrik Falconer**, gynaecological oncologist (Sweden); **Daniela Fanni**, pathologist (Italy); **Angelica Viviana Fletcher**, gynaecological oncologist (Colombia); **Christina Fotopoulou**, gynaecological oncologist (United Kingdom); **Cristina Frutuoso**, gynaecological oncologist (Portugal); **Prafull Ghatage**, gynaecological oncologist (Canada); **Antonio Gil-Moreno**, gynaecological oncologist (Spain); **Frédéric Goffin**, gynaecological oncologist (Belgium); **Francois Golfier**, obstetrician gynaecologist (France); **Mikel Gorostidi**, obstetrician gynaecologist (Spain); **Deborah Gregory**, clinical oncologist (United Kingdom); **Benedetta Guani**, gynaecologist (Switzerland); **Emons Günter**, gynaecological oncologist (Germany); **Frédéric Guyon**, gynaecological oncologist (France); **David Hardisson**, pathologist (Spain); **Annette Hasenburg**, obstetrician gynaecologist (Germany); **Kristina Hellman**, medical oncologist (Sweden); **Gines Hernandez-Cortes**, obstetrician gynaecologist (Spain); **Antonio Herreros**, medical oncologist (Spain); **Peter Hoskin**, clinical oncologist (United Kingdom); **Kim Hulscher**, patient (Netherlands); **Vlora Ibishi**, gynaecologist (Kosovo); **Ahmet Cem Iyibozkurt**, gynaecological oncologist (Turkey); **Nina Boje Kibsgaard Jensen**, clinical oncologist (Denmark); **Kate Johnson**, radiation oncologist (Canada); **Ina Jurgenliemk-Schulz**, radiation oncologist (Netherlands); **Ioannis Kalogiannidis**, gynaecological oncologist (Greece); **Vesna Kesic**, gynaecological oncologist (Serbia); **Pearly Khaw**, radiation oncologist (Australia); **Gurkan Kiran**, gynaecological oncologist (Turkey); **Kathrin Kirchheiner**, radiation oncologist (Austria); **Christian Kirisits**, radiation oncologist (Austria); **Manon Kissel**, radiation oncologist (France); **Marko Klarić**, gynaecological oncologist (Croatia); **Roman Kocian**, gynaecological oncologist (Czech Republic); **Gunnar Kristensen**, gynaecological oncologist (Norway) ; **Kersti Kukk**, gynaecological oncologist (Estonia); **Valentina Lancellotta**, radiation oncologist (Italy); **Fabio Landoni**, gynaecologist (Italy); **Gabriel Lindahl**, gynaecological oncologist (Sweden); **Kristina Loessl**, radiation oncologist (Switzerland); **Tiziano Maggino**, gynaecological oncologist (Italy); **Katarina Majercakova**, radiation oncologist (Spain); **Saadia Mameri**, pathologist (Algeria); **Aljosa Mandic**, gynaecological oncologist (Serbia); **Suzana Manxhuka-Kerliu**, pathologist (Kosovo); **Bogdan Margineanu**, obstetrician gynaecologist (France); **Fabio Martinelli**, gynaecological oncologist (Italy); **Claudia Mateoiu**, pathologist (Sweden); **Xavier Matias-Guiu**, pathologist (Spain); **Mihai Meirovitz**, gynaecological oncologist (Israel); **Eva Meixner**, radiation oncologist (Germany); **Lucas Mendez**, radiation oncologist (Canada); **Miloš Mlynček**, gynaecological oncologist (Slovakia); **David Alejandro Moralez Fernandez**, gynaecological

Cibula D, *et al. Int J Gynecol Cancer* 2023; 33:649–666. doi: 10.1136/ijgc-2023-004429

oncologist (Colombia); **Philippe Morice**, gynaecological oncologist (France); **Esten Nakken**, radiation oncologist (Norway); **Peter Niehoff**, radiation oncologist (Germany); **Eva-Maria Niine-Roolaht**, oncological gynaecologist (Estonia); **Krzysztof Nowosielski**, gynaecological oncologist (Poland); **Ernst Oberlechner**, gynaecological oncologist (Germany); **Claudia Ordeanu**, radiation oncologist (Romania); **Coza Ovidiu Florin**, radiation oncologist (Romania); **Saulius Paskauskas**, gynaecological oncologist (Lithuania); **Anna Myriam Perrone**, gynaecological oncologist (Italy); **Elisabetta Perrucci**, radiation oncologist (Italy); **Patrick Petignat**, obstetrician gynaecologist (Switzerland); **Stamatios Petousis**, gynaecological oncologist (Greece); **Primoz Petric**, radiation oncologist (Switzerland); **Bradley Pieters**, radiation oncologist (Netherlands); **Radovan Pilka**, obstetrician gynaecologist (Czech Republic); **Richard Poetter**, radiation oncologist (Austria); **Mario Preti**, gynaecologist (Italy); **Anna Protasova**, gynaecological oncologist (Russia); **Isabelle Ray-Coquard**, medical oncologist (France); **Nicholas Reed**, clinical oncologist (United Kingdom); **Alexander Reinthaller**, gynaecological oncologist (Austria); **Sophie Renard**, radiation oncologist (France); **Ángeles Rovirosa**, radiation oncologist (Spain); **Vilius Rudaitis**, gynaecological oncologist (Lithuania); **Giovanni Scambia**, gynaecological oncologist (Italy); **Sergio Schettini**, gynaecologist (Italy); **Jalid Sehouli**, gynaecological oncologist (Germany); **Cristiana Sessa**, gynaecological oncologist (Switzerland); **Paul Sevelda**, gynaecological oncologist (Austria); **Philippe Simon**, gynaecological oncologist (Belgium); **Tayup Simsek**, gynaecological oncologist (Turkey); **Piero Sismondi**, obstetrician gynaecologist (Italy); **Tone Skeie-Jensen**, gynaecological oncologist (Norway); **Špela Smrkolj**, gynaecological oncologist (Slovenia); **Erik Soegaard-Andersen**, obstetrician gynaecologist (Denmark); **Sofia Spampinato**, medical physics (Denmark); **Hana Stankusova**, radiation oncologist (Czech Republic); **Simona Stolnicu**, pathologist (Romania); **Eva-Maria Strömsholm**, patient (Finland); **Alina Sturdza**, radiation oncologist (Austria); **Sudha Sundar**, gynaecological oncologist (United Kingdom); **Jacek Jan Sznurkowski**, gynaecological oncologist (Poland); **Li Tee Tan**, clinical oncologist (United Kingdom); **Ekkasit Tharavichitkul**, radiation oncologist (Thailand); **Tayfun Toptas**, gynaecological oncologist (Turkey); **Antonio Travaglino**, pathologist (Italy); **Helen Trihia**, pathologist (Greece); **Elena Ulrikh**, gynaecological oncologist (Russia); **Margit Valgma**, radiation oncologist (Estonia); **Jacobusvan der Velden**, gynaecological oncologist (Netherlands); **Ignace Vergote**, gynaecological oncologist (Belgium); **René Verheijen**, gynaecological oncologist (France); **Lisa Vicenzi**, radiation oncologist (Italy); **Nadia Villena Salinas**, pathologist (Denmark); **Boris Vranes**, gynaecological oncologist (Serbia); **Henrike Westerveld**, radiation oncologist (Netherlands); **Nuri Yildirim**, gynaecological oncologist (Turkey); **Gian Franco Zannoni**, pathologist (Italy).
